# Targeting the ICOS/ICOS‐L pathway in a mouse model of established allergic asthma disrupts T follicular helper cell responses and ameliorates disease

**DOI:** 10.1111/all.13602

**Published:** 2018-11-12

**Authors:** Faith I. Uwadiae, Chloe J. Pyle, Simone A. Walker, Clare M. Lloyd, James A. Harker

**Affiliations:** ^1^ Inflammation, Repair and Development Section National Heart and Lung Institute Imperial College London London UK; ^2^ MRC & Asthma UK Centre in Allergic Mechanisms of Asthma London UK

**Keywords:** allergic airway disease, germinal centres, ICOS, ICOS‐L, T follicular helper cells

## Abstract

**Background:**

Allergic asthma is characterized by chronic inflammation and remodelling of the airways, associated with dysregulated type 2 immune responses and allergen‐specific IgE. T follicular helper cells (T_FH_) are crucial in T‐dependent B‐cell responses and have been implicated in allergic airway disease (AAD). T_FH_, unlike other CD4^+^ T cells, are uniquely reliant on continuous ICOS signalling to maintain their phenotype after T‐cell priming; therefore, disrupting this signal can impair T_FH_ responses. However, the contribution of T_FH_ to disease during chronic aero‐allergen exposure and the therapeutic potential of targeting these cells have not been evaluated.

**Methods:**

To establish AAD, female BALB/c mice were repeatedly exposed to house dust mite or *Alternaria alternata* three times a week for up to 5 weeks. To examine the impact of T_FH_ on AAD, mice were allergen exposed for 5 weeks and co‐administered anti‐ICOS Ligand‐targeted antibodies, three times a week for the last 2 weeks.

**Results:**

T_FH_ were first observed in the lung‐draining lymph nodes and with further exposure were also found locally within the lungs. T_FH_ accumulated with sustained allergen exposure, alongside germinal centre (GC) B cells. Blockade of ICOS signalling after AAD establishment successfully depleted T_FH_ but did not affect the differentiation of other CD4^+^ T‐cell subsets. This reduced GC responses, allergen‐specific IgE, inflammation, pulmonary IL‐13 and airway hyper‐responsiveness.

**Conclusions:**

T_FH_ are crucial in the regulation of AAD and the ICOS/ICOS‐L pathway could represent a novel therapeutic target in allergic asthma.

**Highlights**

- T follicular helper cells (TFH) are first found in the lung draining lymph nodes after 1 week of chronic allergen exposure and with continuous exposure are identified within the lungs
- ICOS‐L blockade during ongoing allergic disease impairs lung and lymph node TFH responses resulting in reduced germinal centre activity and allergen specific IgE
- ICOS‐L blockade also improves pulmonary inflammation and airway hyper‐responsiveness and represents a potential therapeutic intervention during established allergic disease

## 1 INTRODUCTION

Allergic asthma is characterized by chronic inflammation and remodelling of the airways and dysregulated type 2 immune responses. This results from sustained exposure to aeroallergens, including house dust mite (HDM) and fungal spores, which lead to elevated concentrations of type 2 cytokines IL‐4, IL‐5 and IL‐13, alongside allergen‐specific immunoglobulin E (IgE), eosinophilia and airway hyper‐responsiveness (AHR),^1^. T helper 2 cells (Th2), and the type 2 cytokines they produce, have traditionally been thought of as the central drivers of the disease. It is now clear, however, that many other immune cells can produce these cytokines and play vital roles in the regulation of distinct asthma phenotypes.^1^

T_FH_ are a distinct CD4^+^ T‐cell subset specialized to provide help to B cells, resulting in the production of high affinity, isotype‐switched antibodies and the differentiation of B cells into memory B cells and plasma cells.^2^ They can be identified by their expression of CXCR5, PD1, Bcl‐6 and ICOS and are predominantly located within the B‐cell follicles of secondary lymphoid organs (SLOs), such as lymph nodes and the spleen.^2^ T_FH_ differentiation is a multi‐step process. First dendritic cells (DCs) present antigen and co‐stimulatory signals to naive CD4^+^ T cells within the T‐cell zone of SLOs.^3^, ^4^ Next, pre‐T_FH_ migrate towards the T‐B border^5^, ^6^ where antigen presentation and inducible T co‐stimulator ligand (ICOS‐L) co‐stimulation is provided by activated B cells.^7^, ^8^ Fully differentiated T_FH_ migrate into the B‐cell follicle, further migrating into newly formed anatomical structures called germinal centres (GC).^6^ Here, T_FH_ form tight, cognate interactions with GC B cells, providing survival and differentiation signals to the B cells in return for T‐cell receptor (TCR) signalling and co‐stimulation.^2^, ^5^, ^7^ The co‐stimulatory molecule ICOS is required for T‐cell activation and is upregulated on CD4^+^ T cells following TCR engagement.^9^ Importantly, however, T_FH_ uniquely require sustained ICOS/ICOS‐L signalling throughout an immune response and after DC priming to maintain their phenotype, unlike other CD4^+^ T‐cell subsets.^10^

The role of T_FH_ in allergic asthma appears to be complex. T_FH_ are found in the SLOs of mice that have undergone sensitization and challenge with HDM^11^, ^12^, ^13^ and are required for allergen‐specific IgE production.^14^, ^15^ T_FH_ isolated from the lung draining lymph nodes can migrate to the lungs and become Th2 cells, enhancing allergic airway disease (AAD), when injected intravenously.^13^ In contrast, IL‐21^+^ T_FH_ failed to generate Th2 cells when adoptively transferred into naïve mice subsequently challenged with HDM, but do cause airway eosinophillia.^11^ After helminth infection Th2 cells can become T_FH,_ 16 retaining a Th2‐like phenotype, and T_FH_ themselves can obtain effector functions related to other CD4^+^ T‐cell lineages.^17^ Conversely, CD4^+^ T cells lacking the T_FH_ master transcriptional regulator Bcl6, which cannot become T_FH_, preferentially differentiate into lung resident Th2 cells and promote AAD.^12^ Thus, T_FH_ appear to be important in AAD but their exact contribution and the therapeutic potential of targeting them once disease is established are uncertain.

Here, using chronic allergen exposure models of AAD, to mimic the frequent, low dose, allergen exposures allergic asthmatics experience, T_FH_ and GC B cells developed in the SLOs and the lung itself. Targeting the ICOS/ICOS‐L pathway after AAD establishment specifically reduced the frequency of both cell types. Treatment also reduced airway eosinophilia, AHR, allergen‐specific IgE and was associated with reduced concentrations of IL‐13 in the lungs. This shows that blocking ICOS/ICOS‐L interactions during chronic airway inflammation can target T_FH_‐dependent immune responses and ameliorate established AAD.

## 2 MATERIALS AND METHODS

### 2.1 Mice

Six‐ to eight‐week‐old female BALB/c mice were purchased from Charles River Laboratories (UK) and IL‐13^GFP^ reporter mice were gifted by Professor Andrew McKenzie, University of Cambridge. Mice were housed in IVCs, and all procedures were approved by the Imperial College London Animal Welfare Ethical Review Body (AWERB) and the United Kingdom Home Office (Approval from both under project licence number 70/7463) and conducted in accordance with the Animals (Scientific Procedures) Act 1986.

### 2.2 Induction of allergic airway disease and ICOS‐L intervention

Mice were administered 25 μg HDM extract *(Dermatophagoides pteronyssinus)* or 10 μg *Alternaria alternata* (ALT) intranasally (i.n.) three times a week for up to 5 weeks (Greer Laboratories, NC, USA; Citeq, Groningen, The Netherlands). Control mice were given 25 μL PBS. In blocking experiments, from week 4 onwards, mice were co‐administered 150 μg anti‐ICOS‐Ligand (Clone: HK5.3, BioXCell, NH, USA) or isotype control (Clone: 2A3, BioXCell, NH, USA) antibody in 200 μL PBS via intraperitoneal (i.p.) injection three times a week for 2 weeks. Mice were culled at the end of week 5. All animals were harvested 18 hours after the final allergen dose.

### 2.3 Flow cytometry assessment

Cell suspensions were acquired as previously described^18^ and were stained in flow cytometry buffer (PBS containing 2% foetal calf serum and 2 mmol/L EDTA). To reduce nonspecific binding, cell suspensions were incubated with antibody cocktails containing anti‐CD16/32 antibody. Cells were extracellularly stained in antibody cocktails for 30 minutes at 4°C, apart from stains containing CXCR5 which were incubated at room temperature in the dark for 1 hour. For detection of intracellular cytokines, cells were incubated with 50 ng/mL phorbol myristate acetate, 500 ng/mL ionomycin and 10 μg/mL brefeldin A for 5 hours at 37°C and 5% CO_2_. Cells were fixed with 1% paraformaldehyde. For detection of intranuclear transcription factors, cells were fixed and permeabilized using the Foxp3/Transcriptional factor staining buffer set (eBioscience, CA, USA) according to the manufacturer's instructions. Cells were then washed and intracellularly stained at 4°C in permeabilization wash buffer (Biolegend, CA, USA). Flow cytometry data were acquired using an LSRII Fortessa (Becton Dickson, NJ, USA) and analysed using the FlowJo 10 software (FlowJo, OR, USA). Flow cytometry antibodies are listed in Table S1.

### 2.4 Assessment of lung function

Airway hyper‐responsiveness was measured in anesthetized and tracheotomized mice in response to increasing doses of methacholine (3‐100 mg/mL; Sigma‐Aldrich, MO, USA) using the flexiVent system (Scireq, Montreal, Canada) as previously described.^19^

### 2.5 Antibody assessment

Allergen‐specific IgE and IgG1 levels were measured by coating plates with 50 μg/mL HDM then adding serially diluted serum and biotinylated IgG1 or IgE antibodies (BD Pharmingen™, Oxford, UK). Endpoint titre was calculated using baseline+2xSD based on naïve animals.

### 2.6 Cytokine analysis

IL‐13, IL‐17A and IL‐21 were measured using Ready Set Go Kits (eBioscience, CA, USA), Eotaxin‐2 using mouse CCL24/Eotaxin‐2 DuoSet ELISA and IL‐5 using paired antibodies (R&D systems, Abington, UK). All ELISAs were performed according to manufacturer's instructions.

### 2.7 Statistical analysis

Statistical significance was determined using the Mann‐Whitney *U* Test and assessed using Prism 6 (GraphPad Software Inc, CA, USA). All *P* values ≤0.05 (*) ≤0.01 (**), ≤0.001 (***) and ≤0.0001 (****) were considered significant.

Methods continue in Appendix S1.

## 3 RESULTS

### 3.1 Repeated aero‐allergen exposure generates lung‐ and lymphoid‐resident T_FH_

**Figure 1:**
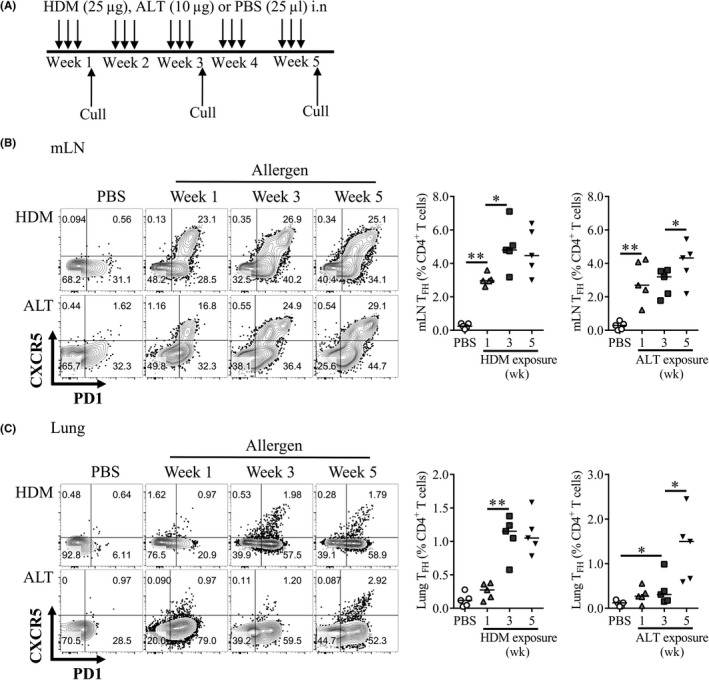
T follicular helper cells (T_FH_) accumulate over time in the mediastinal lymph nodes and lung tissue. Adult female BALB/c mice were exposed to either 25 μg house dust mite (HDM), 10 μg *Alternaria alternata* (ALT) or 25 μL phosphate‐buffered saline (PBS), three times a week for up to 5 weeks. Flow cytometry was used to determine the frequency of T_FH_ within cellular compartments. Representative flow plots of T_FH_ in PBS, ALT or HDM‐treated animals are displayed, pregated on CD4^+^ CD3^+^Foxp3^−^ CD44^hi^ CD62L^−^ lymphocytes. Data are quantified. T_FH_ were defined as CXCR5^+^ PD1^+^Foxp3^−^ CD4^+^ lymphocytes. A, Experimental set‐up. B, mediastinal lymph nodes (mLN), C, lung tissue. Statistical significance was determined using a Mann‐Whitney *U* test. *P < 0.05, **P < 0.01, ***P < 0.001, n = 5 per time‐point. Representative data from two independent experiments

To replicate the repeated low dose aeroallergen exposure experienced by allergic asthmatics, mice were exposed to two common aeroallergens; HDM or ALT three times a week for up to 5 weeks (Figure 1A).

T_FH_, defined as CXCR5^+^PD1^+^Foxp3^−^CD4^+^ (Figure S1) were observed in the lung‐draining mediastinal lymph nodes (mLN) of allergen‐exposed animals after 1 week but were not found in the mLNs of PBS‐treated controls (Figure 1B). Continued allergen exposure further increased T_FH_ proportions in the mLN after 3 and 5 weeks (Figure 1B). T_FH_ were also observed in the spleen but not in the circulation of allergen‐treated animals (Figure S2). Interestingly, T_FH_ were identified in the lung tissue itself after 3 weeks of HDM inhalation and remained elevated at 5 weeks of exposure (Figure 1C). Lung T_FH_ frequencies were also significantly increased following 3 weeks of ALT treatment and further increased after 5 weeks (Figure 1C). Therefore, prolonged allergen exposure induced both local and systemic T_FH_ responses that increased in frequency over time.

### 3.2 T_FH_ precede the development of humoral immunity during AAD

**Figure 2:**
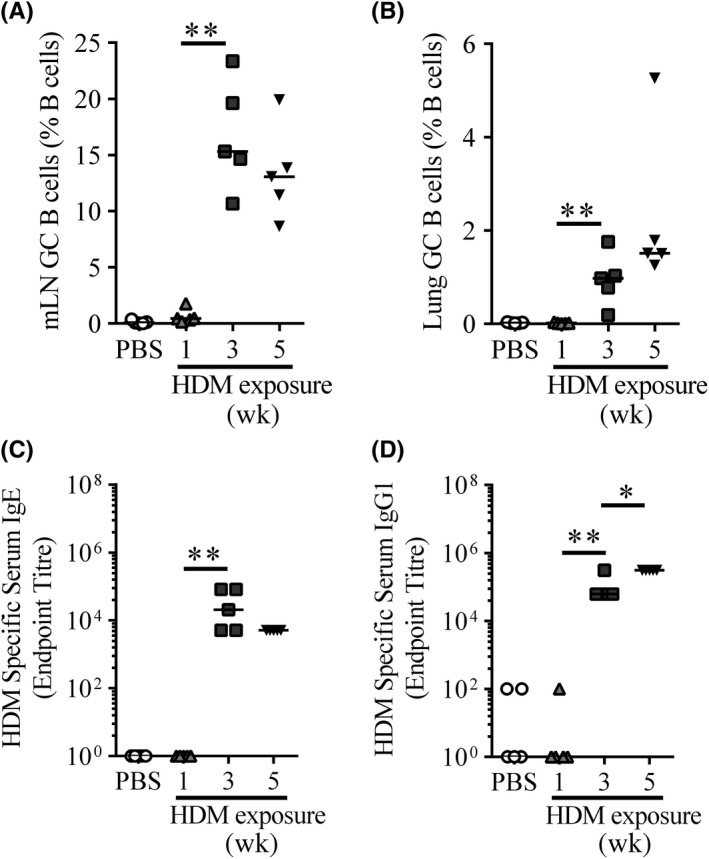
T_FH_ induction precedes germinal centre formation and antibody production. Adult female BALB/c mice were exposed to either 25 μg house dust mite (HDM) or 25 μL phosphate‐buffered saline (PBS), three times a week for up to 5 weeks. A‐B, Flow cytometry was used to determine the frequency of germinal centre (GC) B cells in the mLN and lungs. GC B cell was defined as CD38^−^ GL7^+^ FAS ^+^ CD19^+^B220^+^ lymphocytes and quantified. A, mLN GC B cells, B, Lung GC B cells. C‐D, Serum was titrated, and allergen‐specific IgE and IgG1 were measured by ELISA. Endpoint titres are displayed. C, HDM‐specific IgE, D, HDM‐specific IgG1. Statistical significance was determined using a Mann‐Whitney *U* test. *P < 0.05, **P < 0.01, ***P < 0.001. n = 5 mice per time‐point. Representative data from two independent experiments

T_FH_ direct B‐cell responses, driving GC formation, isotype switching, affinity maturation and B‐cell differentiation.^2^ GC B cells defined as CD38^−^GL7^+^FAS^+^IgD^−^IgM^−^B220^+^CD19^+^ lymphocytes (Figure S3) were absent and comparable to PBS controls in the mLN and lungs after 1 week of aero‐allergen inhalation (Figure 2A and B). However, after 3 weeks of HDM exposure, GC B‐cell frequencies were significantly elevated in the mLN, lungs and spleen, remaining consistently raised between weeks 3 and 5. (Figure 2A, Figure S4). Similar results were observed following ALT exposure (Figure S4).

Allergen‐specific antibodies, especially IgE, are a key feature of AAD. Allergen‐specific IgE and IgG1 were detectable in allergen‐exposed mice from 3 weeks onward (Figure 2C and D). Sustained exposure to HDM did not further alter the levels of allergen‐specific IgE (Figure 2C), but did increase IgG1 (Figure 2D). These data show chronic allergen exposure to generate local and systemic GC B‐cell responses, alongside allergen‐specific antibody which increase over time and are preceded by T_FH_ responses.

### 3.3 ICOS/ICOS‐L interactions sustain T_FH_ and GC B cells

**Figure 3:**
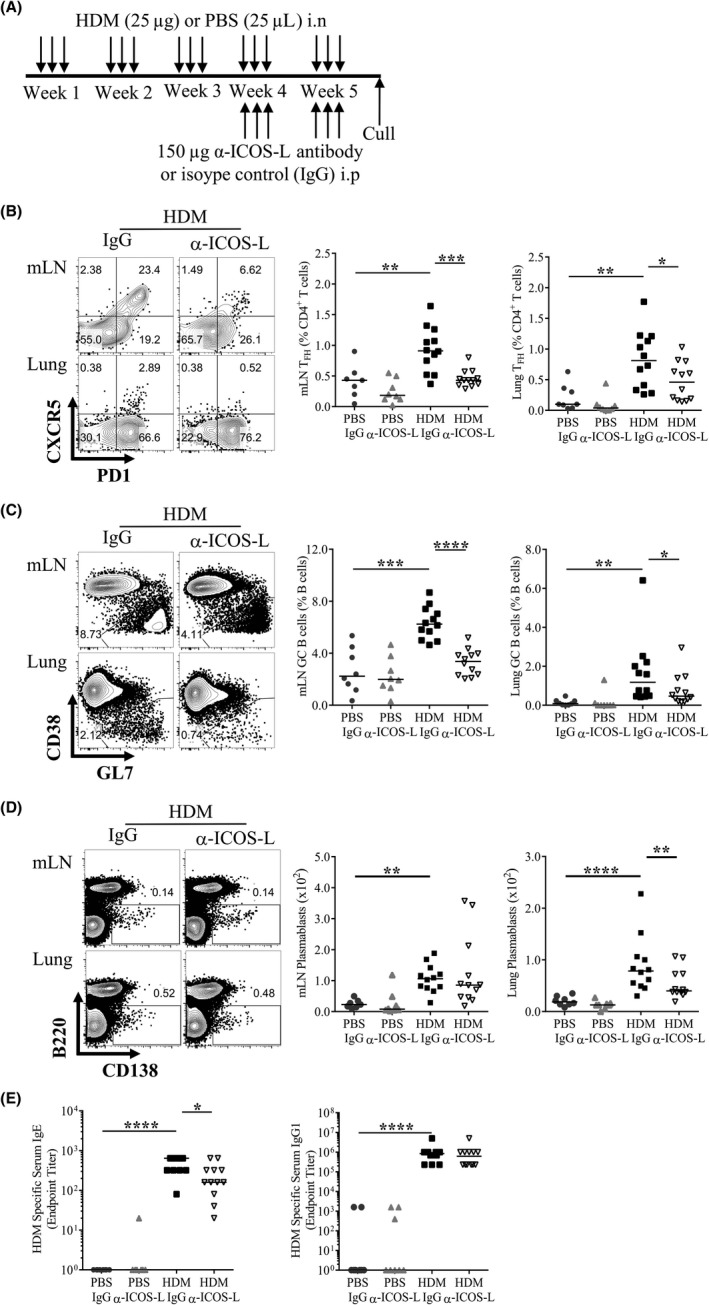
ICOS/ICOS‐L interactions are required to sustain T_FH_ during chronic allergic airway disease (AAD). Adult female BALB/c mice were exposed (i.n) to 25 μg house dust mite (HDM) or 25 μL phosphate‐buffered saline (PBS), three times a week for 5 weeks. From the start of week 4, mice were also administered 150 μg anti‐ICOS‐L (α‐ICOS‐L) or isotype control (IgG) antibody (i.p) three times a week. Mice were culled at the end of week 5. A, Schematic of experimental design, B, Representative flow plots of mLN and lung T_FH_ following HDM and IgG or α‐ICOS‐L treatment. C, Representative flow plots of mLN and lung germinal centre (GC) B cells following HDM and IgG or α‐ICOS‐L treatment. Pre‐gated on CD19^+^B220^+^ B cells. The data are quantified for all groups. D, Representative flow plots of mLN and lung B220^−^ CD138^+^ plasmablasts following HDM and IgG or α‐ICOS‐L treatment, pre‐gated on lymphocytes. Plasmablast numbers are quantified for all groups. E, Serum was titrated, and allergen‐specific antibody was measured by ELISA. Endpoint titres are displayed for IgE and IgG1. Statistical significance was determined using a Mann‐Whitney *U* test. *P < 0.05, **P < 0.01, ***P < 0.001. Data are pooled from two independent experiments, n = 8 for PBS‐treated groups, n = 12 for HDM‐treated groups

T_FH_ require sustained signalling via ICOS to maintain their phenotype and can be depleted by disrupting interactions between ICOS and ICOS‐L.^5^, ^10^ AAD, characterized by AHR and allergic inflammation including humoral immune responses, are established after 3 weeks of allergen exposure.^20^, ^21^ To study the role of ICOS signalling in established AAD, allergen‐treated mice were administered anti‐ICOS‐L antibody (α‐ICOS‐L) or an isotype control (IgG) between weeks 3 and 5 of allergen exposure, and analysed at the end of week 5 (Figure 3A).

α‐ICOS‐L treatment substantially reduced mLN and lung T_FH_ populations after HDM exposure compared to IgG‐treated animals (Figure 3B). Consistent with reduced T_FH_ responses, HDM‐induced mLN and lung GC B cells were also decreased in mice treated with α‐ICOS‐L compared to IgG controls (Figure 3C). α‐ICOS‐L did not decrease the proportion of B220^−^CD138^+^ plasmablasts in the mLN and lungs or the numbers of mLN plasmablasts relative to HDM and IgG‐treated animals but there was a significant reduction in the number of lung plasmablasts (Figure 3D). Furthermore, α‐ICOS‐L intervention diminished serum HDM‐specific IgE but not IgG1 (Figure 3E). Alongside this, α‐ICOS‐L reduced HDM‐induced serum mast cell protease 1 (MCPT1) (Figure S5). Similar results were observed with ALT‐driven AAD (Figures S6 and S7). Overall, these data show that during chronic allergen exposure, T_FH_ can be successfully depleted using α‐ICOS‐L and this is associated with reduced GC B‐cell responses, lung plasmablasts and allergen‐specific IgE but not IgG1.

### 3.4 α‐ICOS‐L treatment dampens lung inflammation and reduces AHR

**Figure 4:**
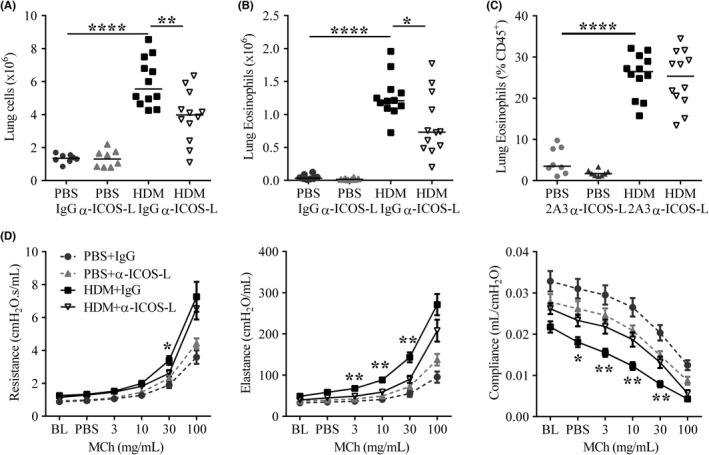
Therapeutic ICOS‐L blockade improves chronic allergic airway disease. Adult female BALB/c mice were exposed (i.n) to 25 μg house dust mite (HDM) or 25 μL phosphate‐buffered saline (PBS), three times a week for 5 weeks. From the start of week 4, mice were also administered 150 μg anti‐ICOS‐L (α‐ICOS‐L) or isotype control (IgG) antibody (i.p) three times a week. Mice were culled at the end of week 5. A, Number of lung cells, B, Lung eosinophil numbers, C, Proportions of lung eosinophils, D, Airway hyper‐responsiveness was measured by exposing mice to 0‐100 mg/mL methacholine (MCh) using the flexiVent system during HDM‐induced allergic airway disease. Airway resistance, elastance and compliance were measured. Curves display mean±SEM. Statistical significance between HDM+ IgG and HDM+α‐ICOS‐L groups was determined using a Mann‐Whitney *U* test. *P < 0.05, **P < 0.01, ***P < 0.001. Data are pooled from two independent experiments, n = 8 for PBS‐treated groups, n = 12 for HDM‐treated groups

Airway hyper‐responsiveness and inflammation are key indicators of AAD progression. α‐ICOS‐L administration successfully reduced aeroallergen induced cellular infiltration into the lungs compared to IgG‐treated controls (Figure 4A). The total number of lung eosinophils was reduced with allergen and α‐ICOS‐L co‐administration (Figure 4B); however, the proportion of lung eosinophils were unchanged (Figure 4C). A similar trend was observed during ALT‐driven AAD (Figure S8).

Allergen‐treated mice showed increased AHR, with raised airway resistance and elastance, and reduced compliance compared to PBS controls in response to increasing doses of methacholine (Figure 4D). Treatment of HDM‐exposed mice with α‐ICOS‐L resulted in reduced airway resistance and elastance and increased compliance, indicative of improved lung function compared to HDM‐treated control mice (Figure 4D). This was also observed for ALT‐induced AAD (Figure S6). Despite this α‐ICOS‐L administration had no impact on HDM‐induced goblet cell hyperplasia, collagen deposition or airway smooth muscle hyperplasia and hypertrophy (Figure S9A‐I). Taken together the data shows that therapeutic administration of α‐ICOS‐L after disease establishment improved airway inflammation and lung function.

### 3.5 α‐ICOS‐L reduces overall inflammation without specifically targeting Th2 cells or ILC2s

**Figure 5:**
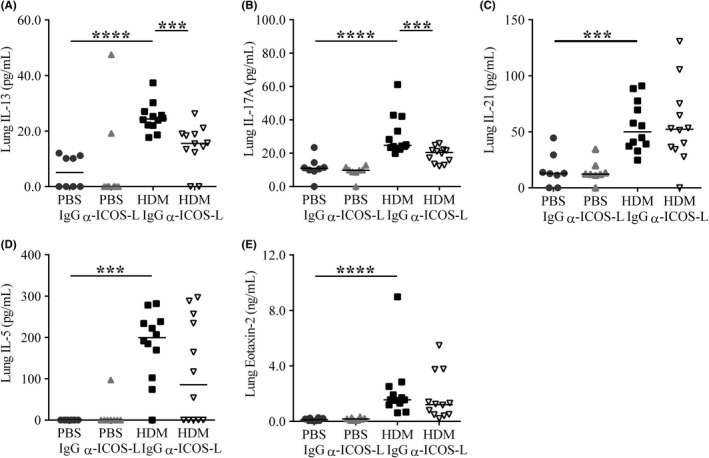
Therapeutic ICOS‐L blockade reduces pulmonary IL‐13 and IL‐17A. Adult female BALB/c mice were exposed (i.n) to 25 μg house dust mite (HDM) or 25 μL phosphate‐buffered saline (PBS), three times a week for 5 weeks. From the start of week 4, mice were also administered 150 μg anti‐ICOS‐L (α‐ICOS‐L) or isotype control (IgG) antibody (i.p) three times a week. Mice were culled at the end of week 5. Pulmonary cytokines and chemokines within lung homogenate were measured by ELISA. A, IL‐13, B, IL‐17A, C, IL‐21, D, IL‐5, E, Eotaxin‐2. Statistical significance was determined using a Mann‐Whitney *U* test. *P < 0.05, **P < 0.01, ***P < 0.001. Data are pooled from two independent experiments, n = 8 for PBS‐treated groups, n = 12 for HDM‐treated groups

To determine the mechanism by which α‐ICOS‐L treatment could be impacting pulmonary inflammation and AHR, the release of several important soluble mediators in the lungs was measured. HDM‐treated animals administered IgG displayed increased lung IL‐13, IL‐17A, IL‐21, IL‐5 and eotaxin‐2 compared to PBS controls (Figure 5A‐E). α‐ICOS‐L treatment reduced lung IL‐13 and IL‐17A concentrations compared to IgG aeroallergen exposed mice (Figure 5A and B). IL‐5 was reduced but did not reach significance, while no change was observed for Eotaxin‐2 (Figure 5D and E). Interestingly, the T_FH_ effector cytokine IL‐21 was unchanged after α‐ICOS‐L treatment (Figure 5C).

**Figure 6:**
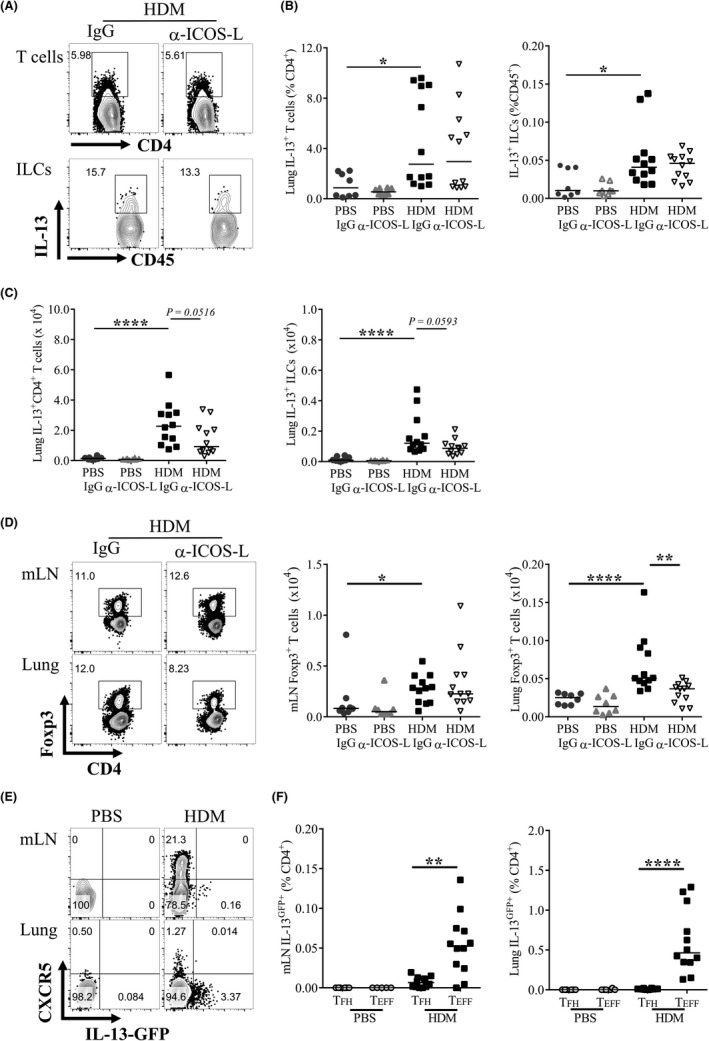
IL‐13^+^ CD4^+^ T cells and ILCs are not directly targeted by ICOS‐L blockade. A‐B, Adult female BALB/c mice were exposed to either 25 μg house dust mite (HDM) or 25 μL phosphate‐buffered saline (PBS), three times a week for up to 5 weeks. From the start of week 4, mice were also administered 150 μg anti‐ICOS‐L (α‐ICOS‐L) or isotype control (IgG) antibody (i.p) three times a week. Mice were culled at the end of week 5. Flow cytometry was used to determine the frequency of lung cellular populations. A, Representative gating of IL‐13^+^ CD4^+^ T cells and IL‐13^+^ ILC2s following HDM and IgG or α‐ICOS‐L treatment. Data are quantified for all groups. B, Proportions of lung IL‐13^+^ T cells and ILCs. C, Numbers of lung IL‐13^+^ T cells and ILCs. Data are pooled from two independent experiments, n = 8 for PBS‐treated groups, n = 12 for HDM‐treated groups. D, Representative flow cytometry of Foxp3^+^ CD4^+^ cells in allergen‐treated mice given IgG or α‐ICOS‐L. Pre‐gated on CD4^+^ CD3^+^ lymphocytes. The number of Foxp3^+^ CD4^+^ T cells is quantified for all groups, E‐F, Adult female IL‐13^GFP^ reporter mice were exposed (i.n) to 25 μg HDM or 25 μL PBS, three times a week for 3 weeks. Flow cytometry was used to determine the frequency of IL‐13^GFP^ cells. E, Representative flow plots of CXCR5^+^ T cells and IL‐13‐GFP ^+^ cells in PBS and HDM‐treated animals, pre‐gated on CD4^+^ CD3^+^ CD44^hi^ CD62L^−^ lymphocytes. F, Quantification of mLN and lung IL‐13^GFP^ ^+^ T_FH_ (CXCR5^+^ PD1^+^ CD4^+^ CD44^hi^ CD62L^−^) and T_EFF_ cells (CXCR5^−^ CD4^+^ CD44^hi^ CD62L^−^). Statistical significance was determined using a Mann‐Whitney *U* test. *P < 0.05, **P < 0.01, ***P < 0.001. Data are pooled from two independent experiments, n = 6 for PBS‐treated groups, n = 12 for HDM‐treated groups

As IL‐13 release was reduced by ICOS‐L blockade and IL‐13 is central to AAD pathogenesis the cellular sources of IL‐13, predominantly thought to be Th2 cells and ILC2s (Lin^−^Nkp46^−^CD45^+^CD90.2^+^IL‐13^+^), were analysed. HDM exposure increased the proportion of IL‐13^+^ CD4^+^ T cells and IL‐13^+^ ILC2s in the lungs, and these were not affected by α‐ICOS‐L intervention (Figure 6A and B). However, consistent with decreased pulmonary inflammation (Figure 4A), total numbers of IL‐13^+^ CD4^+^ T cells and IL‐13^+^ ILC2s showed a trend towards reduction following α‐ICOS‐L treatment (Figure 6C). Although ILC2s are the major ILC subset induced during HDM‐driven chronic AAD,^22^ the total ILC population was also analysed regardless of cytokine expression (Lin^−^Nkp46^−^CD45^+^CD90.2^+.^). Similarly, α‐ICOS‐L did not alter the proportion of total ILCs but did cause a drop in ILC numbers consistent with the overall fall in pulmonary inflammation (Figure S10A). In ALT‐driven AAD, IL‐13^+^ CD4^+^ T cells but not ILC2s were reduced (Figure S10B‐E). Collectively, these data suggest α‐ICOS‐L treatment can therapeutically relieve established AAD by reducing pulmonary inflammation and AHR but does not appear to directly target Th2 cells or ILCs.

IL‐17A^+^ CD4^+^ T cells were also found to be induced following HDM and IgG exposure and again were decreased in total number, but not proportion, after α‐ICOS‐L administration fitting with the fall in overall pulmonary inflammation (Figure S11A‐C). IL‐17A^+^ CD4^+^ T cells were not significantly induced by ALT inhalation and were unchanged by α‐ICOS‐L intervention (Figure S11A, D‐E). IL‐17A^+^ ILCs were unchanged by α‐ICOS‐L intervention (Figure S11A, G‐I).

Regulatory T cells (Tregs) are anti‐inflammatory cells capable of suppressing AAD,^23^ also known to express ICOS. Foxp3^+^ Tregs in the mLNs were unaffected by α‐ICOS‐L intervention both by proportion and by total number compared to HDM‐ and IgG‐treated controls (Figure 6D). Lung Tregs, however, were reduced both in proportion and total number (Figure 6D). Similar results were observed in ALT‐driven AAD (Figure S12). This suggests α‐ICOS‐L may limit lung Foxp3^+^ CD4^+^ T‐cell responses but despite this, the treatment still dampens hallmark features of chronic AAD.

T_FH_ have been shown to accumulate and become dysregulated during sustained antigen exposure,^24^ and to be capable of differentiating into Th2 cells after adoptive transfer.^13^ Here, T_FH_ were reduced together with both secreted IL‐13 and IL‐13^+^ CD4^+^ T cells, therefore the capacity of T_FH_ to produce IL‐13 was examined. Using IL‐13^GFP^ reporter mice, CXCR5 expression was found to be separated from IL‐13^GFP^ expression in CD4^+^ T cells within the mLN and lungs after HDM treatment (Figure 6E and F). IL‐13^GFP+^ cells were, however, readily identified within the non‐T_FH_ T effector (T_EFF_) population (Figure 6E and F). This suggests that T_FH_ are not directly responsible for the increased IL‐13 observed during aeroallergen‐driven AAD or the reduction in IL‐13 observed after α‐ICOS‐L intervention. Overall α‐ICOS‐L treatment was beneficial as a therapeutic during established AAD, targeting both pathogenic humoral immunity intervention and other key features of AAD.

## 4 DISCUSSION

Allergic airway disease is characterized by Th2‐biased lung inflammation and dysregulated humoral immunity in response to recurrent exposure to environmental aero‐allergens.^1^ Here, we examine the importance of T_FH_ in AAD pathology and antibody‐mediated immunity. Chronic allergen exposure results in the development of AAD features, including eosinophilia and IgE. In this context, T_FH_ develop over time, both systemically and locally, and are associated with the presence of GC B cells. Therapeutic targeting of ICOS‐L interrupts the T_FH_ response, decreases humoral immunity and improves hallmark features of AAD.

T_FH_ are initially generated in the peripheral lymph nodes, but with prolonged allergen encounter become detectable locally within the lungs. After acute infection or vaccination, T_FH_ peak between 7 and 14 days declining as antigen availability decreases.^25^, ^26^ In contrast, T_FH_ accumulate over time with repeated allergen exposure, more consistent with other chronic disease models, such as chronic LCMV infection.^27^, ^28^ This aligns with sustained antigen, and therefore continuous TCR stimulation, favouring T_FH_ differentiation and resulting in more T_FH_ in the chronic setting than acute.^24^, ^27^, ^28^ Thereby, highlighting the importance of examining T_FH_ in the context of sustained allergen to replicate the allergic asthmatic experience. Chronic allergen exposure also establishes a local lung T_FH_ population as previously reported by others.^29^ In this study, local T_FH_ are accompanied by lung GC B cells indicative of local lymphoid structures. Thus mimicking the isolated lymphoid cell clusters found at increased frequencies in the lungs of asthmatics compared to healthy controls which have been implicated in pathology.^30^

T_FH_ require continuous ICOS/ICOS‐L signalling after priming to maintain their phenotype.^10^ Therefore, common variable immunodeficiency patients with ICOS deficiency fail to generate T_FH,_ 31 while ICOS overexpression and thus T_FH_ accumulation occurs in *Roquin* mutant mice, which are unable to degrade ICOS.^32^ Inhibiting ICOS/ICOS‐L interactions after initial T‐cell activation has been widely used in several acute models to specifically deplete T_FH_ when antigen is limiting.^5^, ^10^, ^24^ Here, we show that late therapeutic administration of α‐ICOS‐L during ongoing chronic inflammation, when antigen is readily available, can also sufficiently reduce both T_FH_ resident in SLOs and tertiary lymphoid tissue.

Consistent with T_FH_ reductions after ICOS‐L blockade GC B cells in the lungs and SLOs and lung plasmablasts are also reduced. Even after this relatively short intervention HDM‐specific IgE, but not IgG1, also decline. IgE's half‐life is short in comparison to other immunoglobulin isotypes^33^; therefore, after serum transfer Der p1‐ and Lol p 1‐specific IgE decline rapidly over a 50‐day period while IgG remains stable.^34^ Consequently, a more prolonged α−ICOS‐L protocol may be required to sufficiently alter IgG1.^21^ Nonetheless serum MCPT1, an important indicator of mast cell activation,^35^ also decreases suggesting the observed IgE reduction to adequately affect the inflammatory response. As T_FH_ are required for antibody generation including allergen‐specific IgE,^14^, ^15^ and we observe reduced allergen‐specific IgE even after a short intervention, this highlights the potential of transiently targeting T_FH_ to abrogate IgE‐mediated clinical features not only in AAD but also in other IgE‐mediated diseases.

Along with its’ effects on antibody‐mediated immunity targeting the ICOS/ICOS‐L pathway after disease establishment improves allergen‐driven AHR and pulmonary inflammation. IL‐13 potently induces AHR^36^, ^37^ and in consort HDM‐induced pulmonary IL‐13 is reduced after α‐ICOS‐L treatment. Previous studies show T_FH_ to be the only CD4^+^ T‐cell subset affected by late α‐ICOS‐L intervention.^5^, ^10^ During AAD, T_FH_ depletion does not appear to be directly responsible for IL‐13 reductions as AAD‐induced T_FH_ do not acquire a “T_FH_2” phenotype and produce IL‐13.^38^ Instead, the predominant IL‐13 source in the lungs is ILC2s and Th2 cells. ILC2s express ICOS and are dependent on ICOS/ICOS‐L for their homeostatic survival and AAD initiating functions^39^; however, we find α‐ICOS‐L treatment after AAD initiation to not significantly affect IL‐13^+^ ILCs, suggesting ILC2s to not require continuous ICOS signalling to mediate their function. Th2 cells decrease in number after ICOS‐L blockade, along with the overall fall in gross cellular inflammation, but not by proportion, suggesting a limited role for ICOS signalling in maintaining Th2 cell differentiation. Similarly, α‐ICOS‐L treatment slightly affects lung Treg proportions, and significantly reduces numbers after AAD. Despite the role of Tregs in suppressing AAD,^23^ α‐ICOS‐L treatment reduces overall disease severity, suggesting the effect on Tregs to occur as a result of reduced inflammation.

This suggests ICOS signalling after initial priming is required to maintain T_FH_, but not ILC2 nor Th2 cells. Furthermore, this implies the dominant effect of ICOS‐L blockade in AAD, beyond reducing GC activity and antibody‐mediated immunity, is to dampen inflammatory immune responses. Therapeutic blockade of ICOS‐L specifically reduce GC formation in the lung, and this in itself may be sufficient to limit lung inflammation. However, it remains likely that α‐ICOS‐L alters the activity of other immune cells in vivo to reduce inflammation*,* although not sufficiently for us to measure an effect ex vivo in this study. Critically, irrespective of mechanism, α‐ICOS‐L administration shows significant therapeutic potential.

Previous work blocking ICOS or ICOS‐L has been done prophylactically, during disease inception^40^, ^41^, ^42^ or prior to an exacerbation^40^ using less clinically relevant ovalbumin‐induced AAD models,^40^, ^41^, ^42^ which are difficult to deliver clinically. Here, using a more realistic ongoing exposure model and clinically relevant aeroallergens, we show α‐ICOS‐L to reduce disease at the height of pathology. Additionally, we show for the first time in AAD that the prevailing outcome of therapeutic ICOS blockade is not only depletion of T_FH_ and their associated GCs, but also improvements in multiple disease facets. This approach could, therefore, be complimentary or advantageous to currently approved biological therapies, such as omalizumab (anti‐IgE mAb), reslizumab (anti‐IL‐5 mAb) and mepolizumab (IL‐5 antagonist), which generally favour one arm of the allergic response and thus are only effective in specific asthma endotypes.^43^ Furthermore, blocking ICOS signalling has been proven to be safe and effective in phase I clinical trials for systemic lupus erthymatoesus.^44^

Overall, we show T_FH_ arise progressively during aero‐allergen exposure, first in the lymph nodes and with prolonged exposure in the lungs themselves. Even during chronic allergen exposure, T_FH_ are reliant on ICOS‐L signalling for their maintenance and this pathway can be targeted to therapeutically deplete T_FH_ and their downstream effects during chronic disease. This approach reduces inflammation and AHR and implicates T_FH_ as a pathogenic cell type during sustained allergen exposure.

## CONFLICTS OF INTEREST

The authors declare that they have no conflicts of interest.

## AUTHOR CONTRIBUTIONS

F.I.U, J.A.H and C.M.L designed experiments. F.I.U, J.A.H, C.J.P and S.A.W carried out the experimental work. F.I.U analysed the experimental work. F.I.U wrote the manuscript. J.A.H and C.M.L provided feedback.
