# Supplementary material for: Targeting the ICOS/ICOS‐L pathway in a mouse model of established allergic asthma disrupts T follicular helper cell responses and ameliorates disease
Source: Allergy. 2018 Nov 12;74(4):650–62. doi: 10.1111/all.13602 (PMC6492018; doi:10.1111/all.13602)
Supplement: Supplementary file 1 [file ALL-74-650-s001.pptx]

## Slide 1
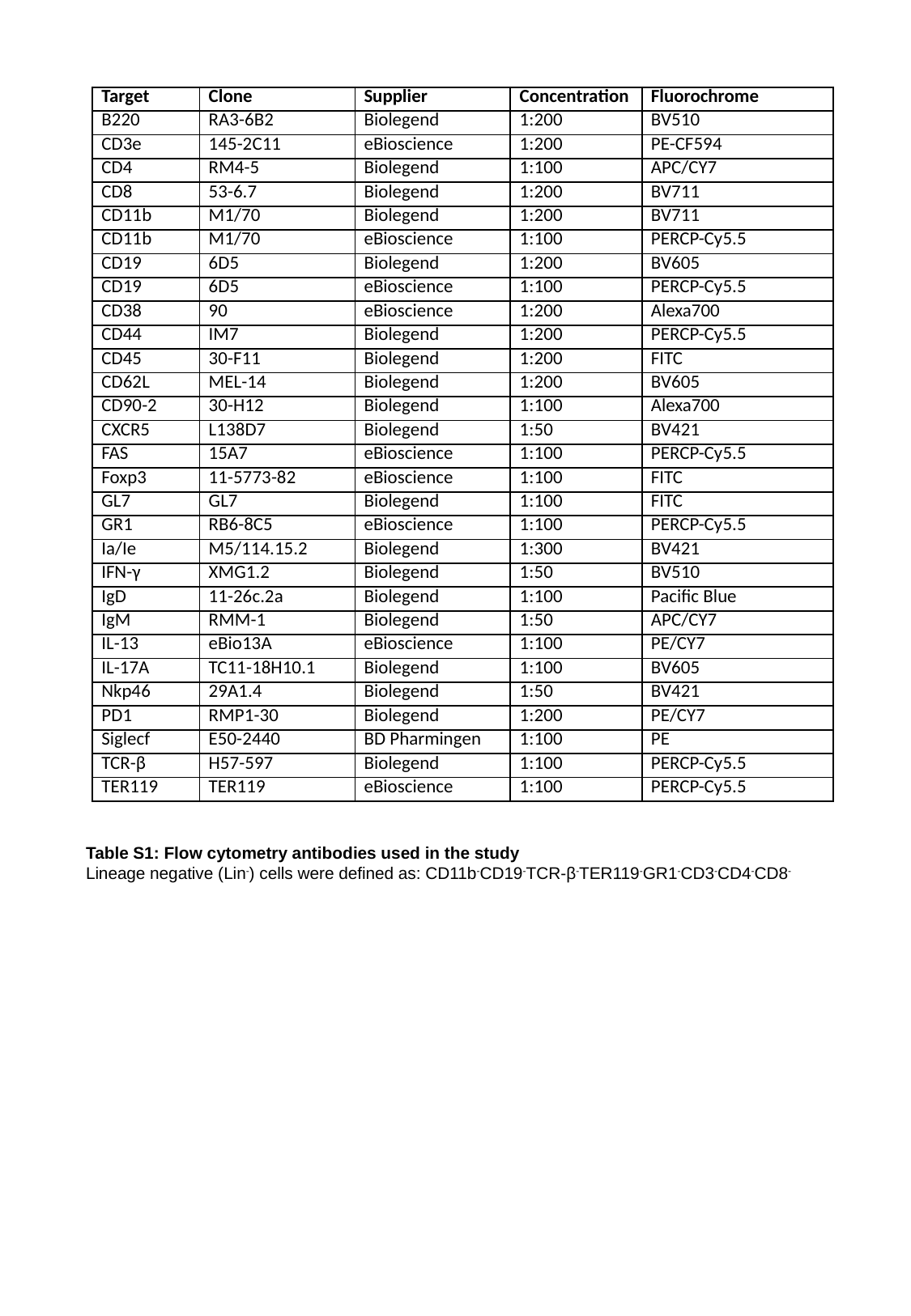

| Target | Clone | Supplier | Concentration | Fluorochrome |
| --- | --- | --- | --- | --- |
| B220 | RA3-6B2 | Biolegend | 1:200 | BV510 |
| CD3e | 145-2C11 | eBioscience | 1:200 | PE-CF594 |
| CD4 | RM4-5 | Biolegend | 1:100 | APC/CY7 |
| CD8 | 53-6.7 | Biolegend | 1:200 | BV711 |
| CD11b | M1/70 | Biolegend | 1:200 | BV711 |
| CD11b | M1/70 | eBioscience | 1:100 | PERCP-Cy5.5 |
| CD19 | 6D5 | Biolegend | 1:200 | BV605 |
| CD19 | 6D5 | eBioscience | 1:100 | PERCP-Cy5.5 |
| CD38 | 90 | eBioscience | 1:200 | Alexa700 |
| CD44 | IM7 | Biolegend | 1:200 | PERCP-Cy5.5 |
| CD45 | 30-F11 | Biolegend | 1:200 | FITC |
| CD62L | MEL-14 | Biolegend | 1:200 | BV605 |
| CD90-2 | 30-H12 | Biolegend | 1:100 | Alexa700 |
| CXCR5 | L138D7 | Biolegend | 1:50 | BV421 |
| FAS | 15A7 | eBioscience | 1:100 | PERCP-Cy5.5 |
| Foxp3 | 11-5773-82 | eBioscience | 1:100 | FITC |
| GL7 | GL7 | Biolegend | 1:100 | FITC |
| GR1 | RB6-8C5 | eBioscience | 1:100 | PERCP-Cy5.5 |
| Ia/Ie | M5/114.15.2 | Biolegend | 1:300 | BV421 |
| IFN-γ | XMG1.2 | Biolegend | 1:50 | BV510 |
| IgD | 11-26c.2a | Biolegend | 1:100 | Pacific Blue |
| IgM | RMM-1 | Biolegend | 1:50 | APC/CY7 |
| IL-13 | eBio13A | eBioscience | 1:100 | PE/CY7 |
| IL-17A | TC11-18H10.1 | Biolegend | 1:100 | BV605 |
| Nkp46 | 29A1.4 | Biolegend | 1:50 | BV421 |
| PD1 | RMP1-30 | Biolegend | 1:200 | PE/CY7 |
| Siglecf | E50-2440 | BD Pharmingen | 1:100 | PE |
| TCR-β | H57-597 | Biolegend | 1:100 | PERCP-Cy5.5 |
| TER119 | TER119 | eBioscience | 1:100 | PERCP-Cy5.5 |
Table S1: Flow cytometry antibodies used in the study
Lineage negative (Lin-) cells were defined as: CD11b-CD19-TCR-β-TER119-GR1-CD3-CD4-CD8-

## Slide 2
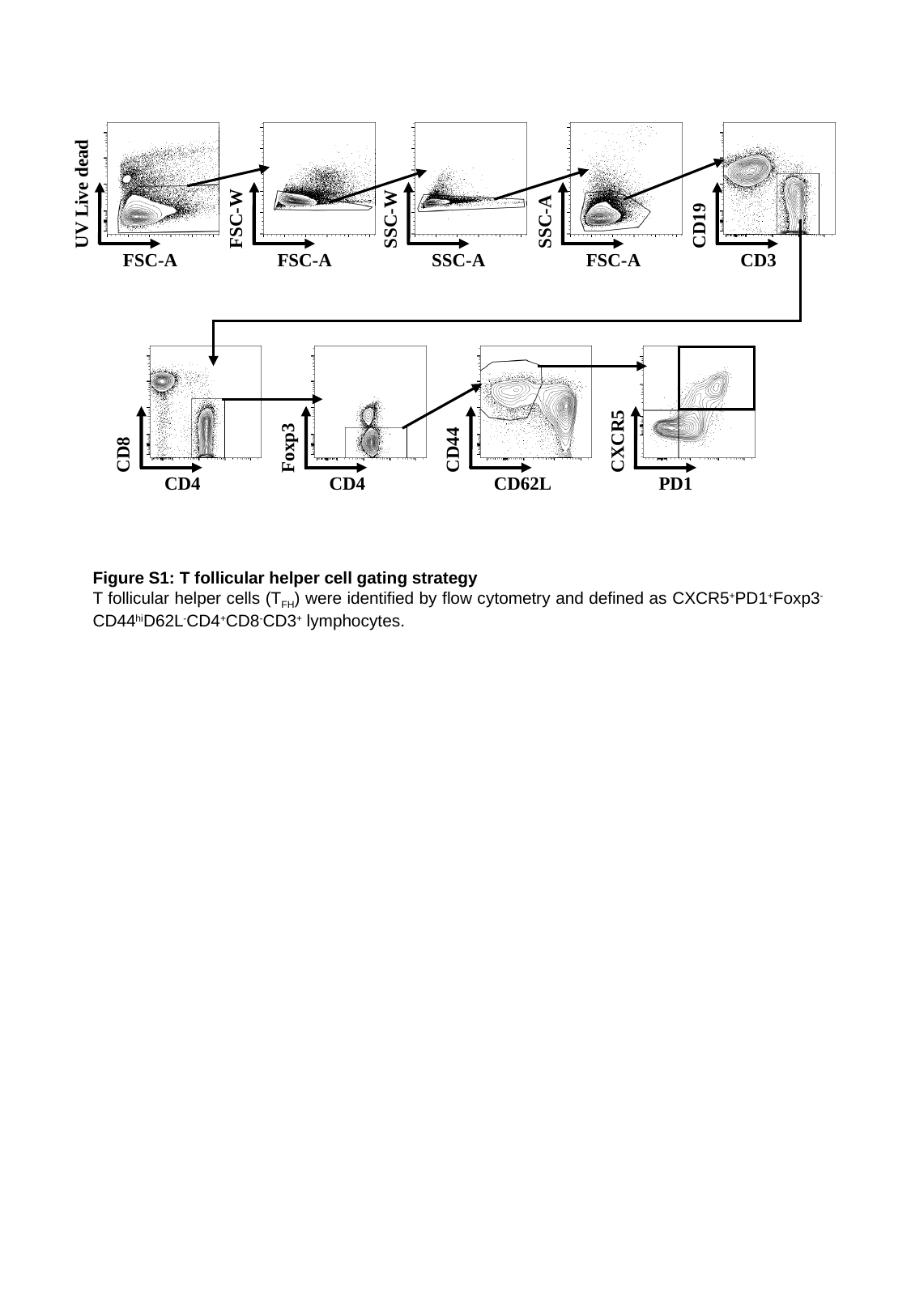

UV Live dead
FSC-A
FSC-W
FSC-A
SSC-W
SSC-A
SSC-A
FSC-A
CD19
CD3
CD8
CD4
Foxp3
CD4
CD44
CD62L
CXCR5
PD1
Figure S1: T follicular helper cell gating strategy
T follicular helper cells (TFH) were identified by flow cytometry and defined as CXCR5+PD1+Foxp3-CD44hiD62L-CD4+CD8-CD3+ lymphocytes.

## Slide 3
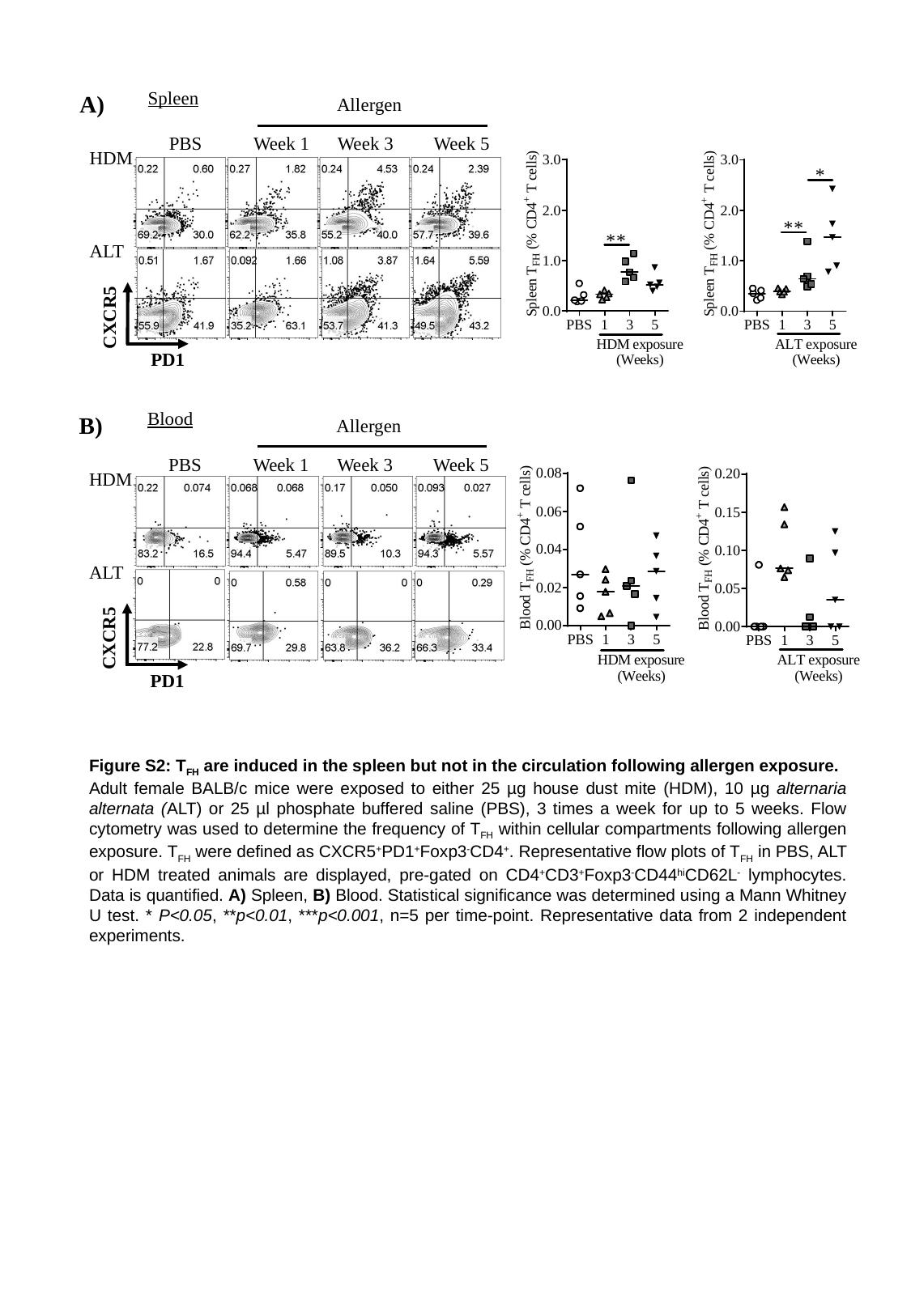

Allergen
Spleen
HDM
PBS
Week 1
Week 3
ALT
CXCR5
PD1
A)
Week 5
Blood
B)
Allergen
HDM
PBS
Week 1
Week 3
Week 5
ALT
CXCR5
PD1
Figure S2: TFH are induced in the spleen but not in the circulation following allergen exposure.
Adult female BALB/c mice were exposed to either 25 µg house dust mite (HDM), 10 µg alternaria alternata (ALT) or 25 µl phosphate buffered saline (PBS), 3 times a week for up to 5 weeks. Flow cytometry was used to determine the frequency of TFH within cellular compartments following allergen exposure. TFH were defined as CXCR5+PD1+Foxp3-CD4+. Representative flow plots of TFH in PBS, ALT or HDM treated animals are displayed, pre-gated on CD4+CD3+Foxp3-CD44hiCD62L- lymphocytes. Data is quantified. A) Spleen, B) Blood. Statistical significance was determined using a Mann Whitney U test. * P<0.05, **p<0.01, ***p<0.001, n=5 per time-point. Representative data from 2 independent experiments.

## Slide 4
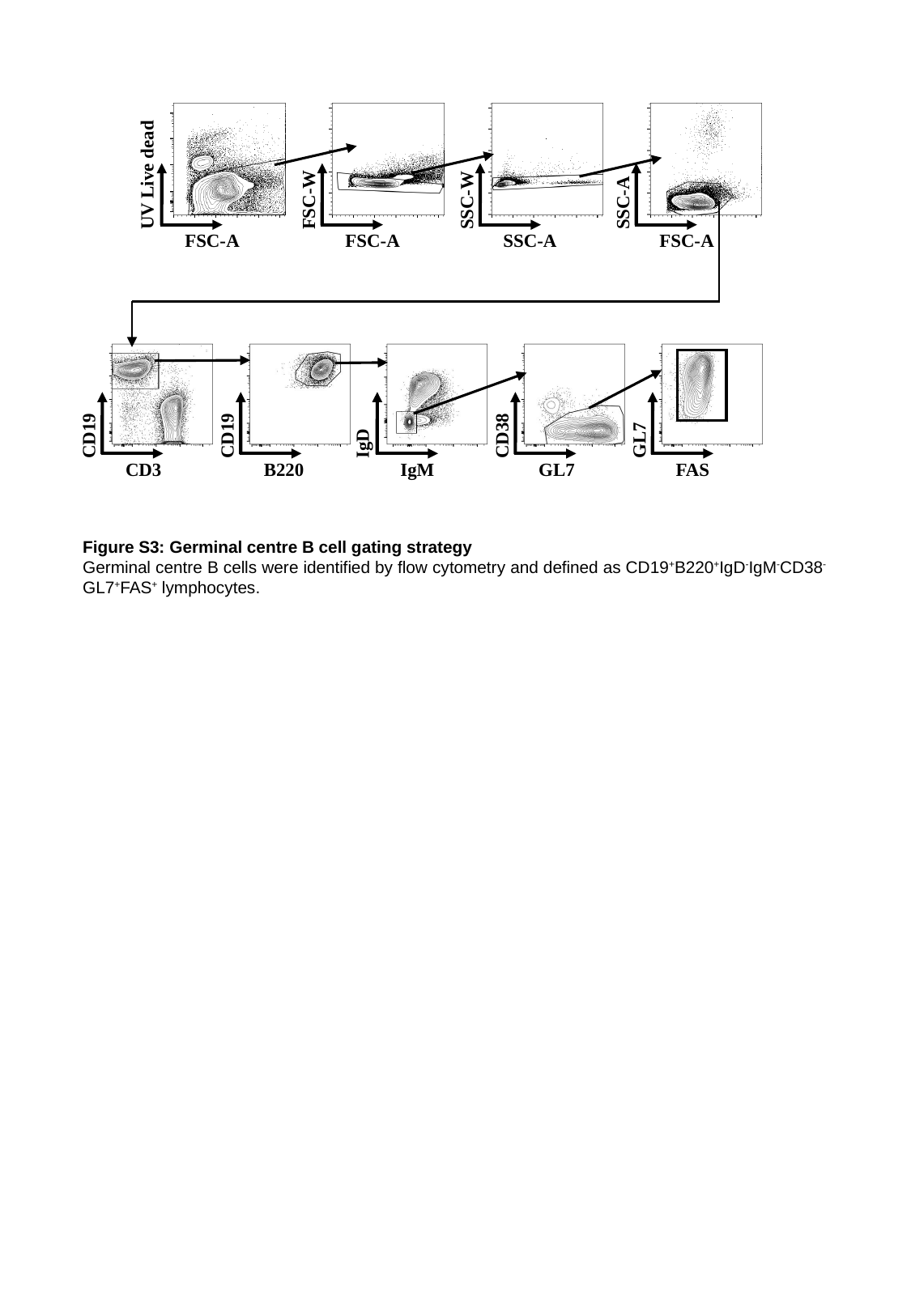

UV Live dead
FSC-A
FSC-W
FSC-A
SSC-W
SSC-A
SSC-A
FSC-A
CD19
CD3
CD19
B220
IgD
IgM
CD38
GL7
GL7
FAS
Figure S3: Germinal centre B cell gating strategy
Germinal centre B cells were identified by flow cytometry and defined as CD19+B220+IgD-IgM-CD38-GL7+FAS+ lymphocytes.

## Slide 5
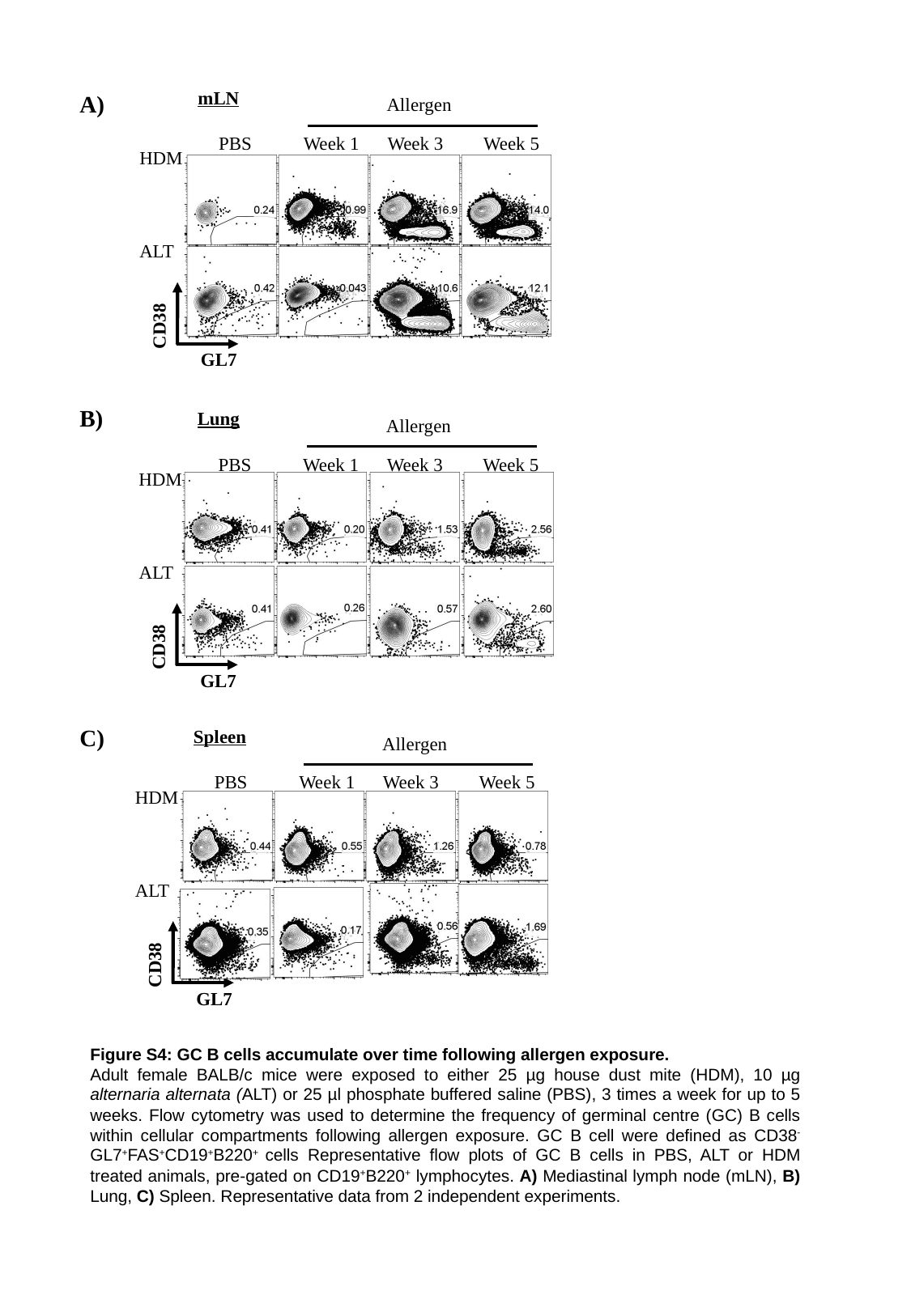

mLN
Allergen
HDM
PBS
Week 1
Week 3
Week 5
ALT
CD38
GL7
A)
Lung
B)
Allergen
HDM
PBS
Week 1
Week 3
Week 5
ALT
CD38
GL7
C)
Spleen
Allergen
HDM
PBS
Week 1
Week 3
Week 5
ALT
CD38
GL7
Figure S4: GC B cells accumulate over time following allergen exposure.
Adult female BALB/c mice were exposed to either 25 µg house dust mite (HDM), 10 µg alternaria alternata (ALT) or 25 µl phosphate buffered saline (PBS), 3 times a week for up to 5 weeks. Flow cytometry was used to determine the frequency of germinal centre (GC) B cells within cellular compartments following allergen exposure. GC B cell were defined as CD38-GL7+FAS+CD19+B220+ cells Representative flow plots of GC B cells in PBS, ALT or HDM treated animals, pre-gated on CD19+B220+ lymphocytes. A) Mediastinal lymph node (mLN), B) Lung, C) Spleen. Representative data from 2 independent experiments.

## Slide 6
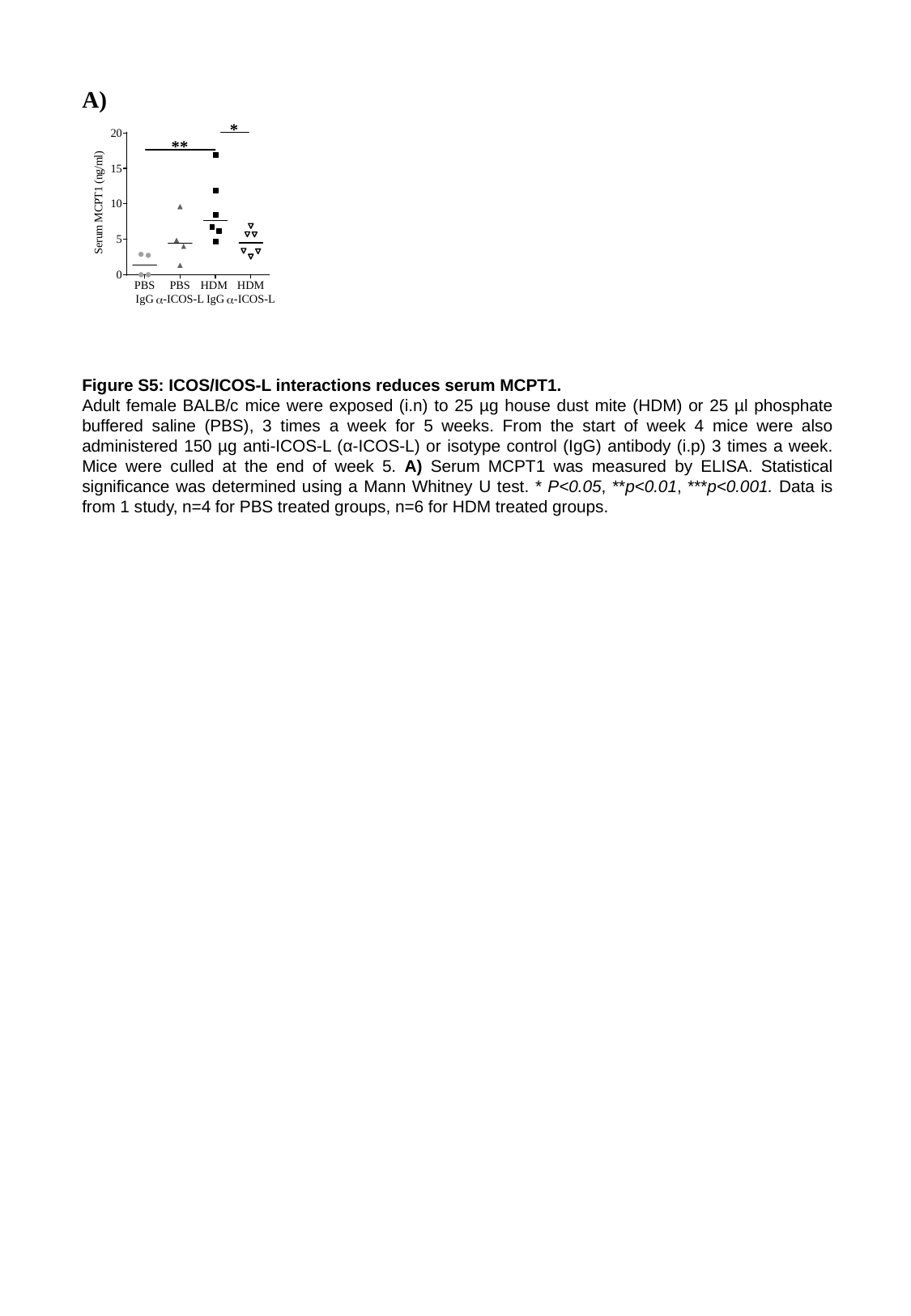

A)
Figure S5: ICOS/ICOS-L interactions reduces serum MCPT1.
Adult female BALB/c mice were exposed (i.n) to 25 µg house dust mite (HDM) or 25 µl phosphate buffered saline (PBS), 3 times a week for 5 weeks. From the start of week 4 mice were also administered 150 µg anti-ICOS-L (α-ICOS-L) or isotype control (IgG) antibody (i.p) 3 times a week. Mice were culled at the end of week 5. A) Serum MCPT1 was measured by ELISA. Statistical significance was determined using a Mann Whitney U test. * P<0.05, **p<0.01, ***p<0.001. Data is from 1 study, n=4 for PBS treated groups, n=6 for HDM treated groups.

## Slide 7
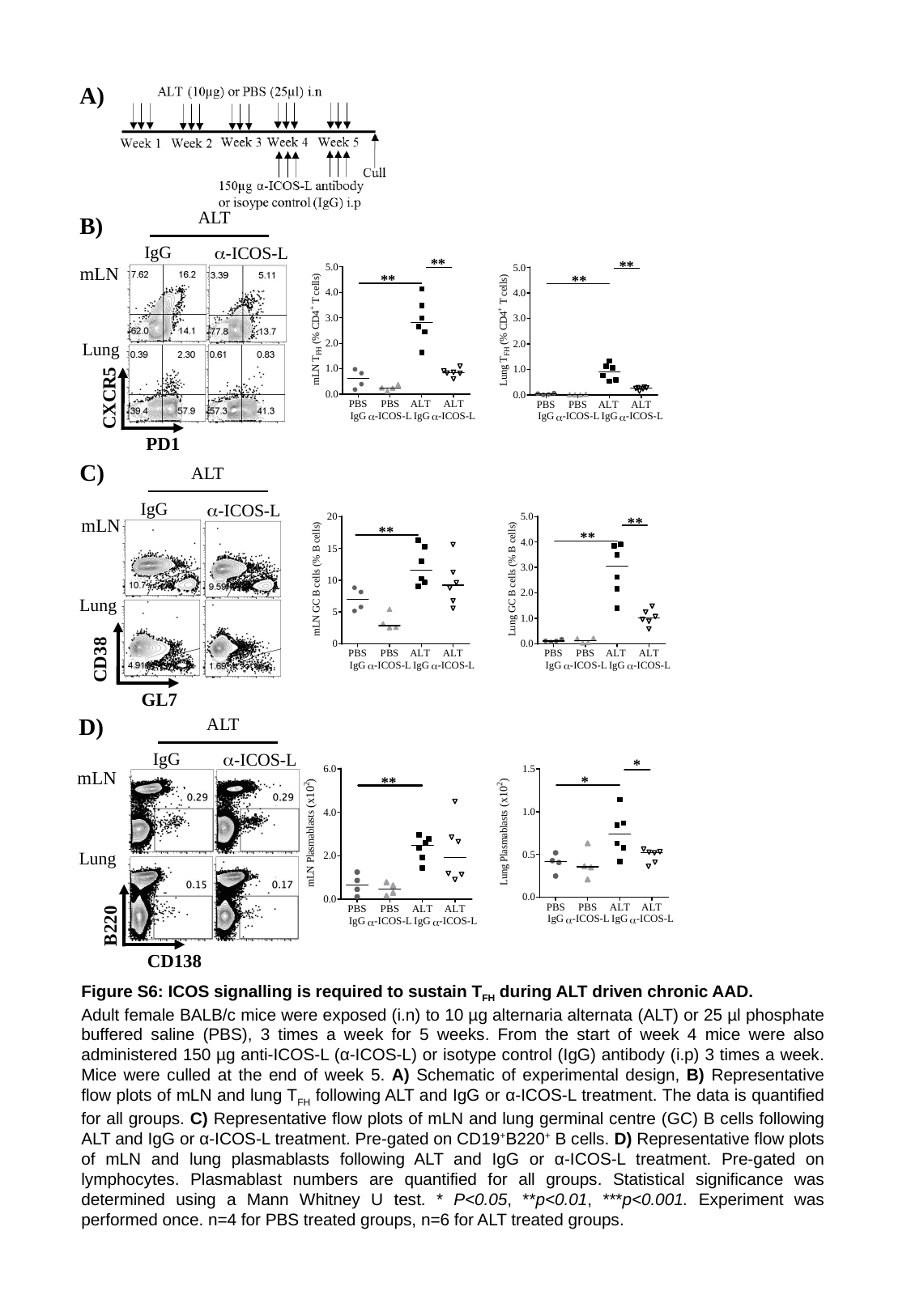

A)
ALT
IgG
a-ICOS-L
mLN
Lung
CXCR5
PD1
B)
C)
ALT
IgG
a-ICOS-L
mLN
Lung
CD38
GL7
D)
ALT
IgG
a-ICOS-L
mLN
Lung
B220
CD138
Figure S6: ICOS signalling is required to sustain TFH during ALT driven chronic AAD.
Adult female BALB/c mice were exposed (i.n) to 10 µg alternaria alternata (ALT) or 25 µl phosphate buffered saline (PBS), 3 times a week for 5 weeks. From the start of week 4 mice were also administered 150 µg anti-ICOS-L (α-ICOS-L) or isotype control (IgG) antibody (i.p) 3 times a week. Mice were culled at the end of week 5. A) Schematic of experimental design, B) Representative flow plots of mLN and lung TFH following ALT and IgG or α-ICOS-L treatment. The data is quantified for all groups. C) Representative flow plots of mLN and lung germinal centre (GC) B cells following ALT and IgG or α-ICOS-L treatment. Pre-gated on CD19+B220+ B cells. D) Representative flow plots of mLN and lung plasmablasts following ALT and IgG or α-ICOS-L treatment. Pre-gated on lymphocytes. Plasmablast numbers are quantified for all groups. Statistical significance was determined using a Mann Whitney U test. * P<0.05, **p<0.01, ***p<0.001. Experiment was performed once. n=4 for PBS treated groups, n=6 for ALT treated groups.

## Slide 8
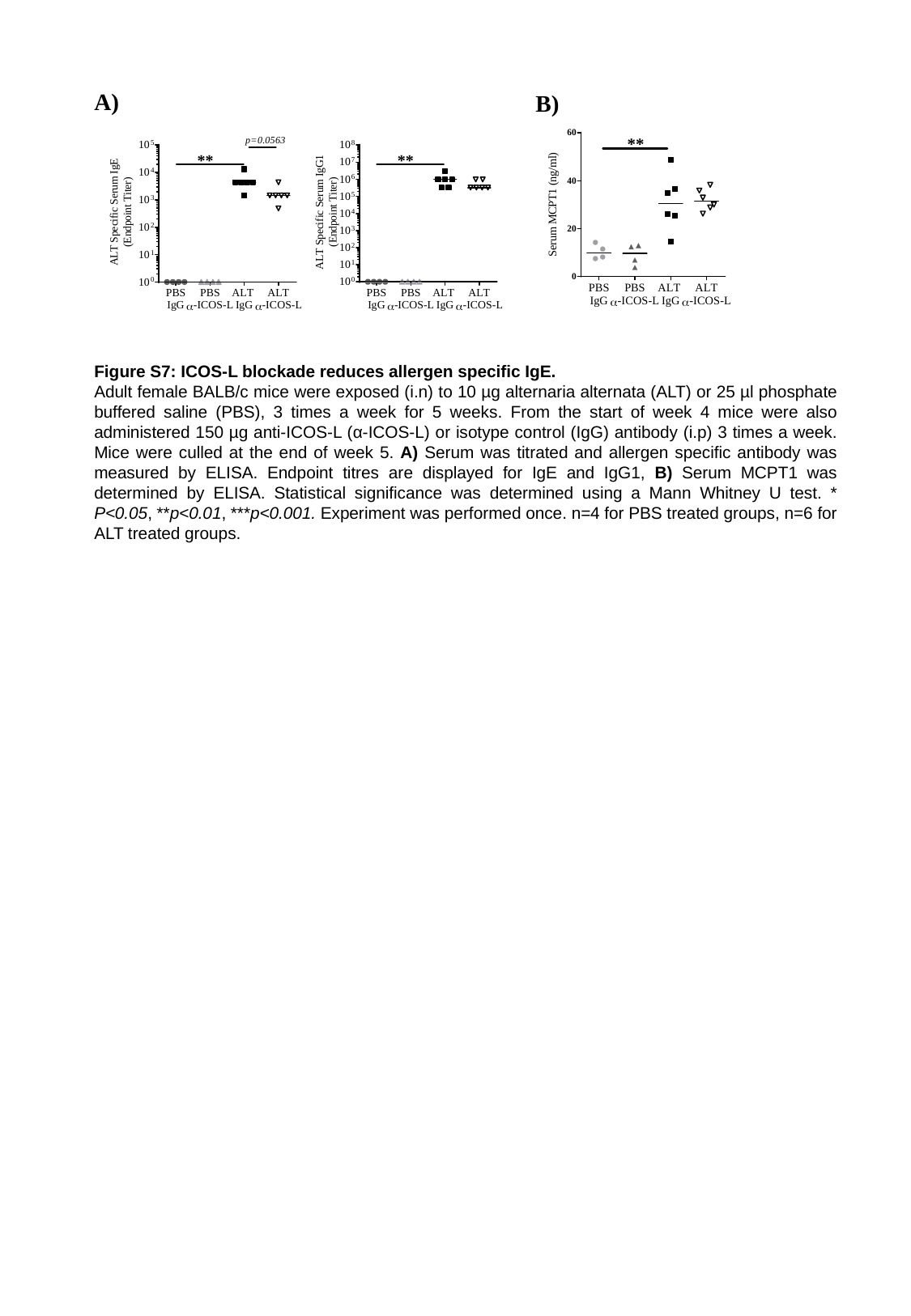

A)
B)
Figure S7: ICOS-L blockade reduces allergen specific IgE.
Adult female BALB/c mice were exposed (i.n) to 10 µg alternaria alternata (ALT) or 25 µl phosphate buffered saline (PBS), 3 times a week for 5 weeks. From the start of week 4 mice were also administered 150 µg anti-ICOS-L (α-ICOS-L) or isotype control (IgG) antibody (i.p) 3 times a week. Mice were culled at the end of week 5. A) Serum was titrated and allergen specific antibody was measured by ELISA. Endpoint titres are displayed for IgE and IgG1, B) Serum MCPT1 was determined by ELISA. Statistical significance was determined using a Mann Whitney U test. * P<0.05, **p<0.01, ***p<0.001. Experiment was performed once. n=4 for PBS treated groups, n=6 for ALT treated groups.

## Slide 9
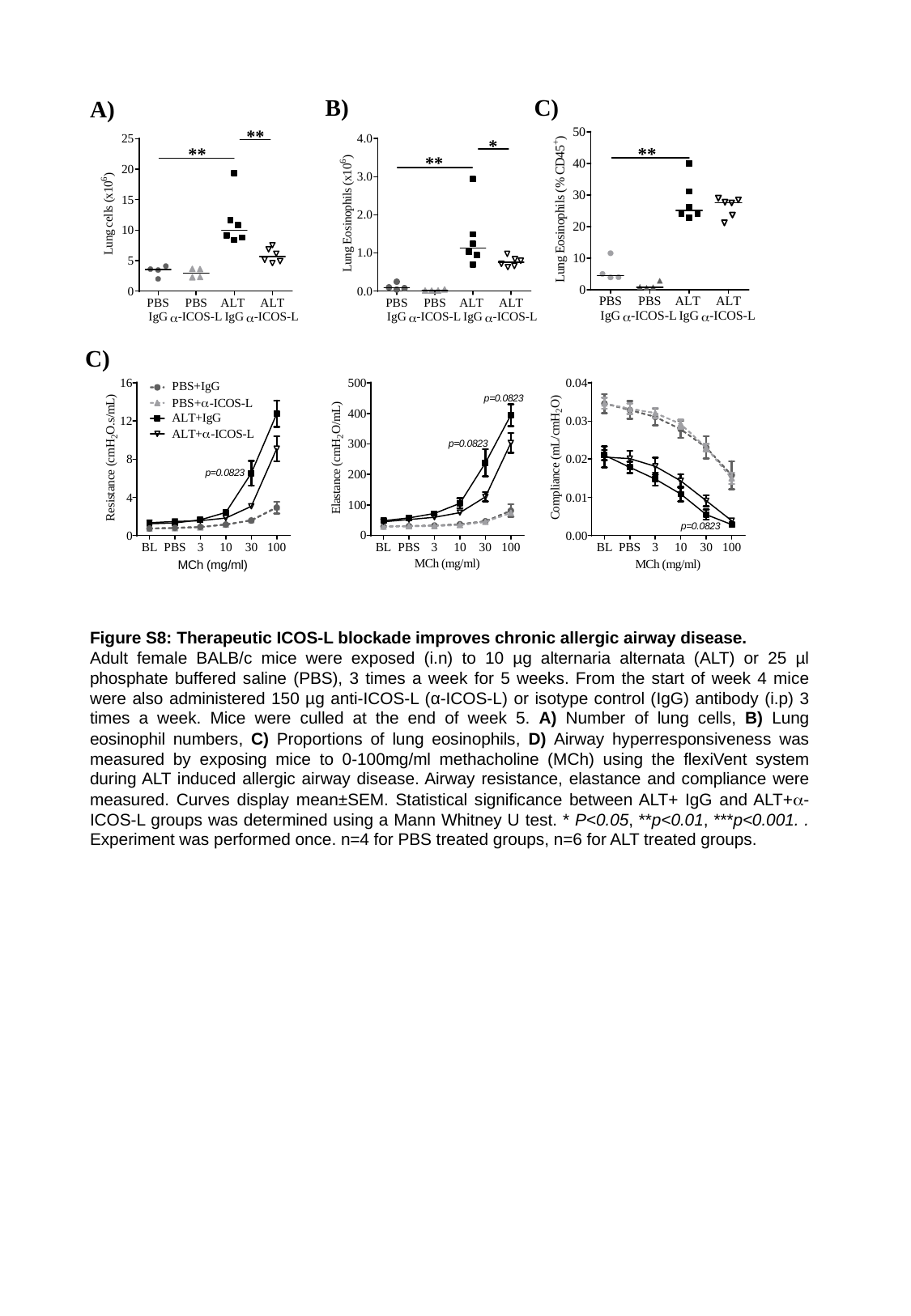

B)
C)
A)
C)
Figure S8: Therapeutic ICOS-L blockade improves chronic allergic airway disease.
Adult female BALB/c mice were exposed (i.n) to 10 µg alternaria alternata (ALT) or 25 µl phosphate buffered saline (PBS), 3 times a week for 5 weeks. From the start of week 4 mice were also administered 150 µg anti-ICOS-L (α-ICOS-L) or isotype control (IgG) antibody (i.p) 3 times a week. Mice were culled at the end of week 5. A) Number of lung cells, B) Lung eosinophil numbers, C) Proportions of lung eosinophils, D) Airway hyperresponsiveness was measured by exposing mice to 0-100mg/ml methacholine (MCh) using the flexiVent system during ALT induced allergic airway disease. Airway resistance, elastance and compliance were measured. Curves display mean±SEM. Statistical significance between ALT+ IgG and ALT+a-ICOS-L groups was determined using a Mann Whitney U test. * P<0.05, **p<0.01, ***p<0.001. . Experiment was performed once. n=4 for PBS treated groups, n=6 for ALT treated groups.

## Slide 10
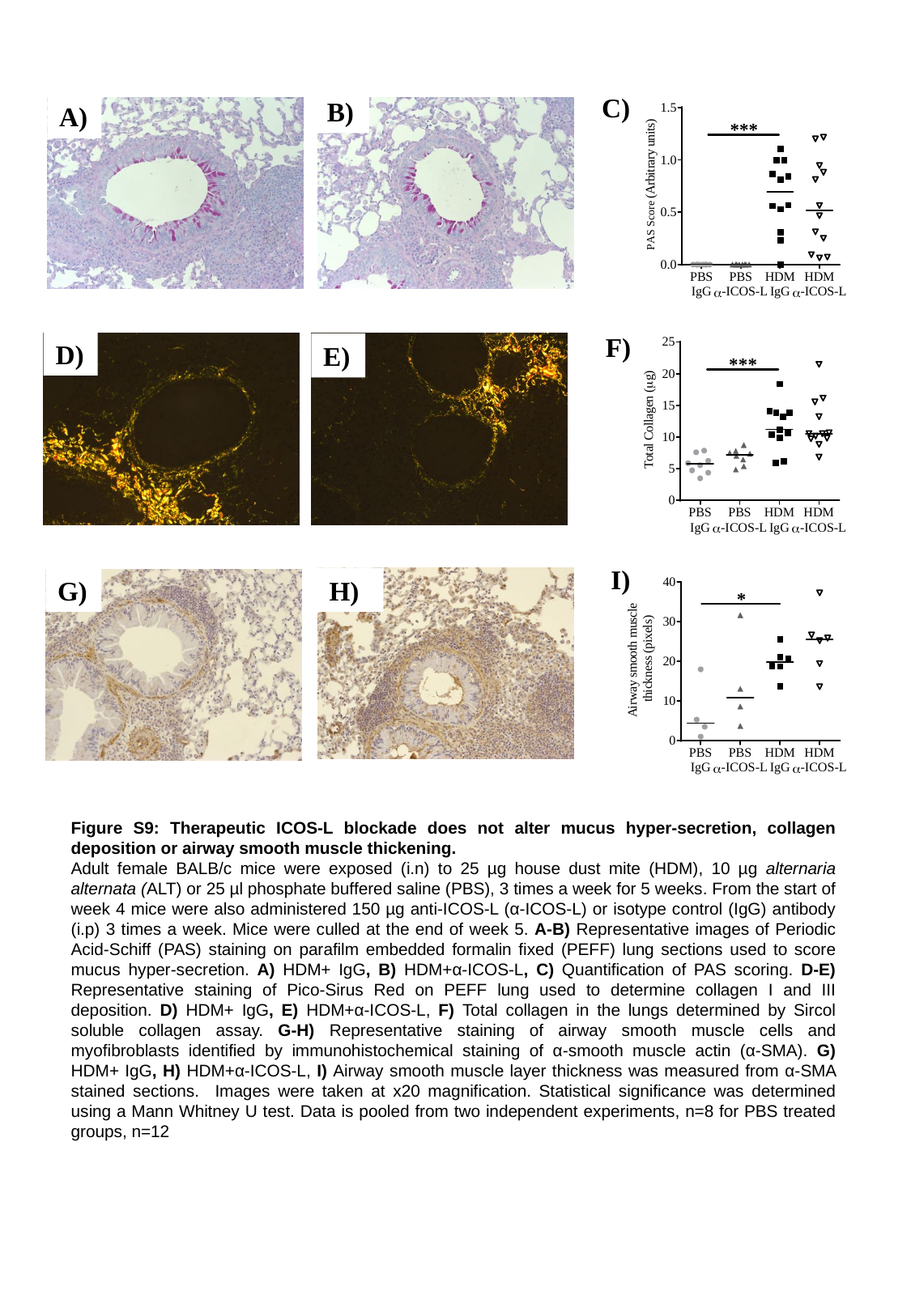

C)
B)
A)
F)
D)
E)
I)
G)
H)
Figure S9: Therapeutic ICOS-L blockade does not alter mucus hyper-secretion, collagen deposition or airway smooth muscle thickening.
Adult female BALB/c mice were exposed (i.n) to 25 µg house dust mite (HDM), 10 µg alternaria alternata (ALT) or 25 µl phosphate buffered saline (PBS), 3 times a week for 5 weeks. From the start of week 4 mice were also administered 150 µg anti-ICOS-L (α-ICOS-L) or isotype control (IgG) antibody (i.p) 3 times a week. Mice were culled at the end of week 5. A-B) Representative images of Periodic Acid-Schiff (PAS) staining on parafilm embedded formalin fixed (PEFF) lung sections used to score mucus hyper-secretion. A) HDM+ IgG, B) HDM+α-ICOS-L, C) Quantification of PAS scoring. D-E) Representative staining of Pico-Sirus Red on PEFF lung used to determine collagen I and III deposition. D) HDM+ IgG, E) HDM+α-ICOS-L, F) Total collagen in the lungs determined by Sircol soluble collagen assay. G-H) Representative staining of airway smooth muscle cells and myofibroblasts identified by immunohistochemical staining of α-smooth muscle actin (α-SMA). G) HDM+ IgG, H) HDM+α-ICOS-L, I) Airway smooth muscle layer thickness was measured from α-SMA stained sections. Images were taken at x20 magnification. Statistical significance was determined using a Mann Whitney U test. Data is pooled from two independent experiments, n=8 for PBS treated groups, n=12

## Slide 11
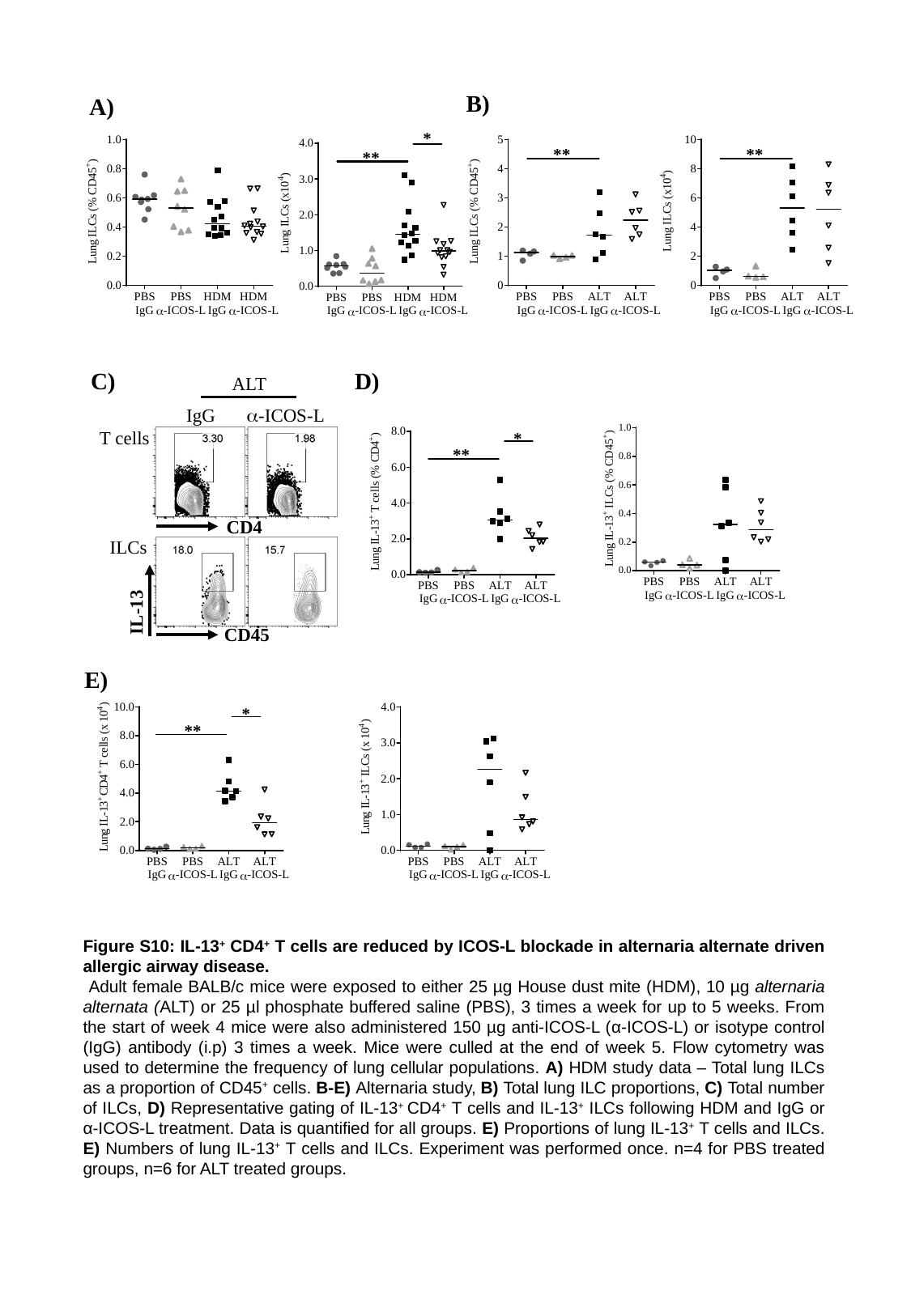

B)
A)
C)
D)
ALT
IgG
a-ICOS-L
T cells
CD4
ILCs
IL-13
CD45
E)
Figure S10: IL-13+ CD4+ T cells are reduced by ICOS-L blockade in alternaria alternate driven allergic airway disease.
 Adult female BALB/c mice were exposed to either 25 µg House dust mite (HDM), 10 µg alternaria alternata (ALT) or 25 µl phosphate buffered saline (PBS), 3 times a week for up to 5 weeks. From the start of week 4 mice were also administered 150 µg anti-ICOS-L (α-ICOS-L) or isotype control (IgG) antibody (i.p) 3 times a week. Mice were culled at the end of week 5. Flow cytometry was used to determine the frequency of lung cellular populations. A) HDM study data – Total lung ILCs as a proportion of CD45+ cells. B-E) Alternaria study, B) Total lung ILC proportions, C) Total number of ILCs, D) Representative gating of IL-13+ CD4+ T cells and IL-13+ ILCs following HDM and IgG or α-ICOS-L treatment. Data is quantified for all groups. E) Proportions of lung IL-13+ T cells and ILCs. E) Numbers of lung IL-13+ T cells and ILCs. Experiment was performed once. n=4 for PBS treated groups, n=6 for ALT treated groups.

## Slide 12
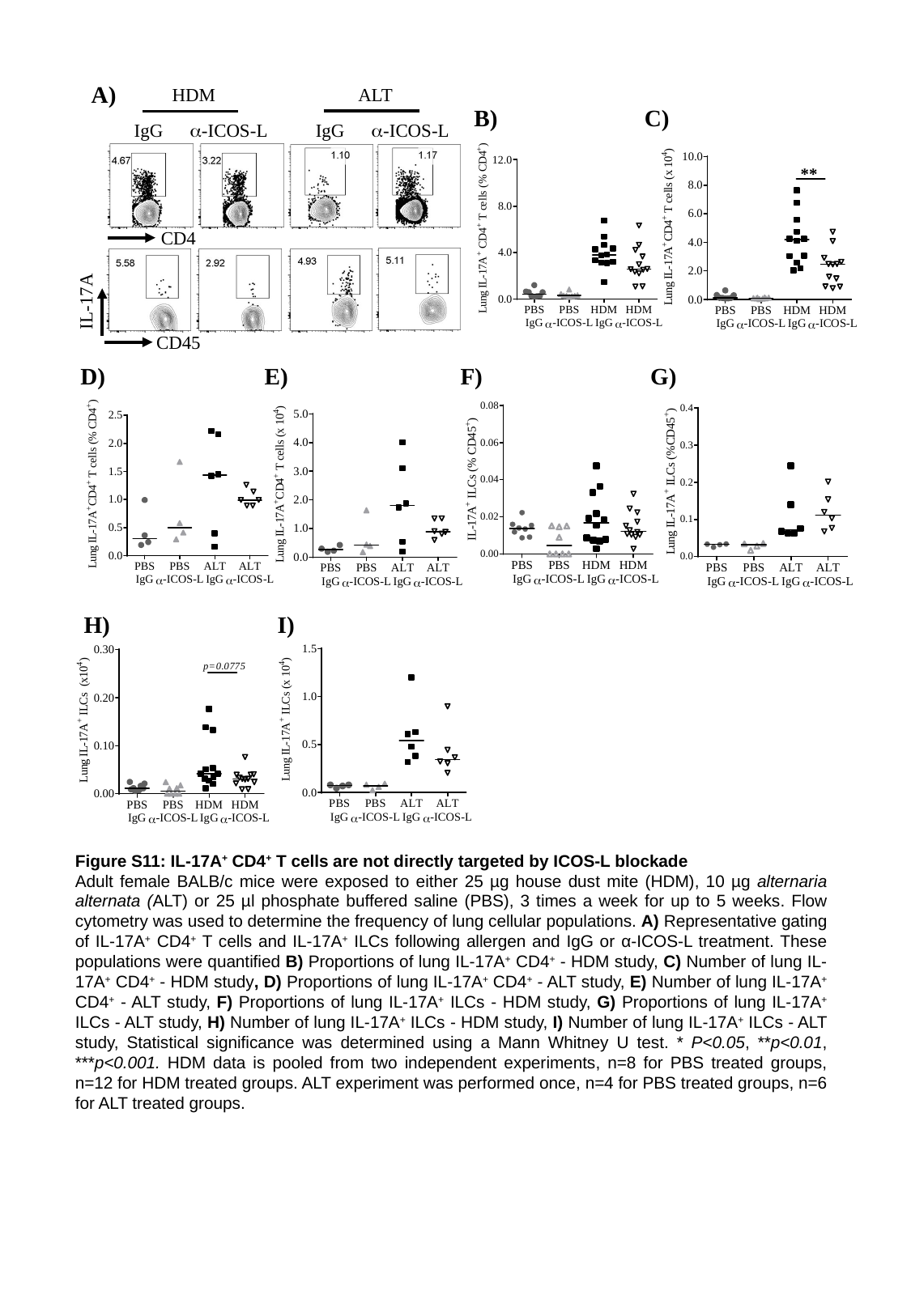

A)
ALT
HDM
IgG
a-ICOS-L
IgG
a-ICOS-L
CD4
CD45
IL-17A
B)
C)
D)
E)
F)
G)
H)
I)
Figure S11: IL-17A+ CD4+ T cells are not directly targeted by ICOS-L blockade
Adult female BALB/c mice were exposed to either 25 µg house dust mite (HDM), 10 µg alternaria alternata (ALT) or 25 µl phosphate buffered saline (PBS), 3 times a week for up to 5 weeks. Flow cytometry was used to determine the frequency of lung cellular populations. A) Representative gating of IL-17A+ CD4+ T cells and IL-17A+ ILCs following allergen and IgG or α-ICOS-L treatment. These populations were quantified B) Proportions of lung IL-17A+ CD4+ - HDM study, C) Number of lung IL-17A+ CD4+ - HDM study, D) Proportions of lung IL-17A+ CD4+ - ALT study, E) Number of lung IL-17A+ CD4+ - ALT study, F) Proportions of lung IL-17A+ ILCs - HDM study, G) Proportions of lung IL-17A+ ILCs - ALT study, H) Number of lung IL-17A+ ILCs - HDM study, I) Number of lung IL-17A+ ILCs - ALT study, Statistical significance was determined using a Mann Whitney U test. * P<0.05, **p<0.01, ***p<0.001. HDM data is pooled from two independent experiments, n=8 for PBS treated groups, n=12 for HDM treated groups. ALT experiment was performed once, n=4 for PBS treated groups, n=6 for ALT treated groups.

## Slide 13
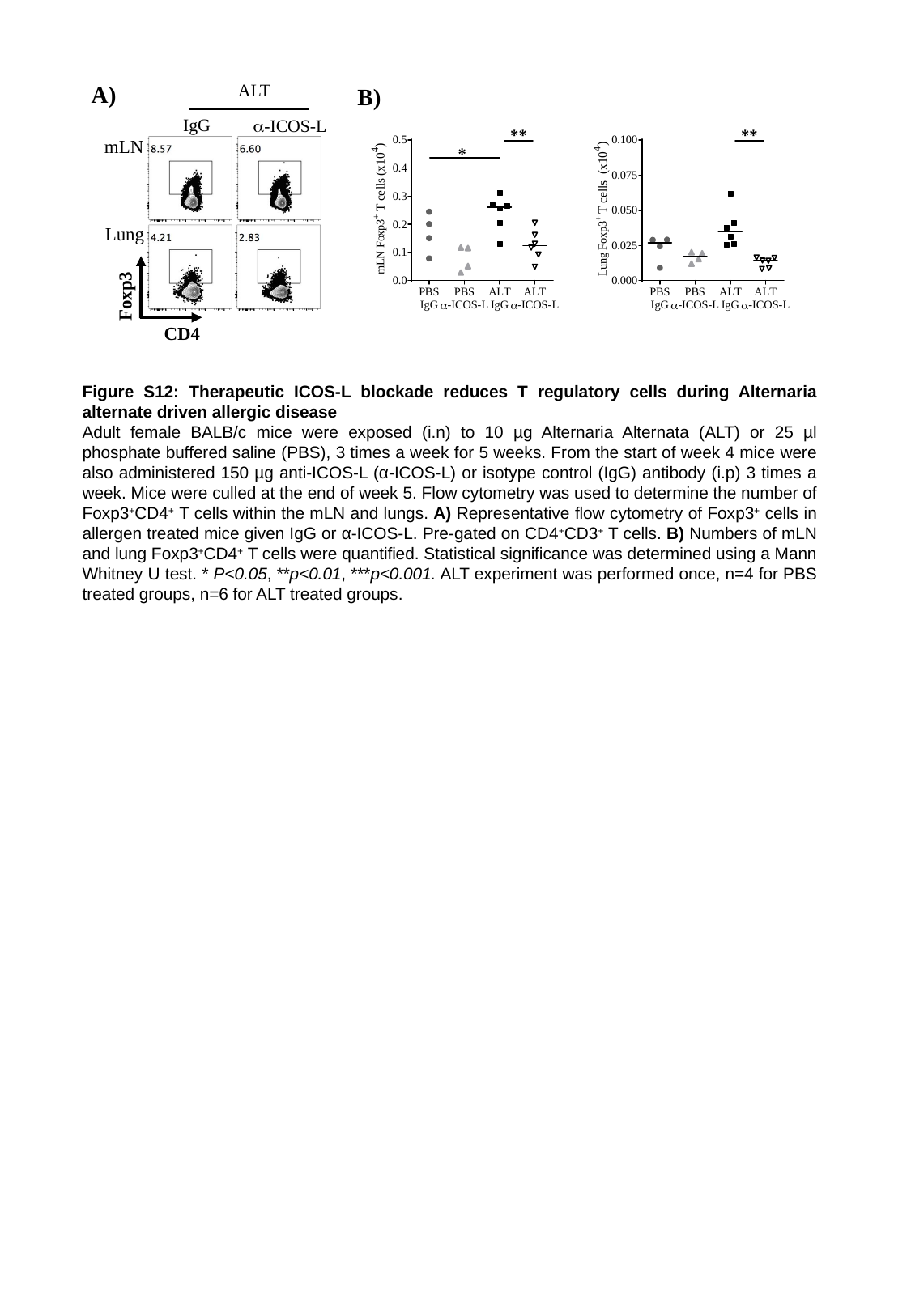

A)
ALT
IgG
a-ICOS-L
mLN
Foxp3
CD4
Lung
B)
Figure S12: Therapeutic ICOS-L blockade reduces T regulatory cells during Alternaria alternate driven allergic disease
Adult female BALB/c mice were exposed (i.n) to 10 µg Alternaria Alternata (ALT) or 25 µl phosphate buffered saline (PBS), 3 times a week for 5 weeks. From the start of week 4 mice were also administered 150 µg anti-ICOS-L (α-ICOS-L) or isotype control (IgG) antibody (i.p) 3 times a week. Mice were culled at the end of week 5. Flow cytometry was used to determine the number of Foxp3+CD4+ T cells within the mLN and lungs. A) Representative flow cytometry of Foxp3+ cells in allergen treated mice given IgG or α-ICOS-L. Pre-gated on CD4+CD3+ T cells. B) Numbers of mLN and lung Foxp3+CD4+ T cells were quantified. Statistical significance was determined using a Mann Whitney U test. * P<0.05, **p<0.01, ***p<0.001. ALT experiment was performed once, n=4 for PBS treated groups, n=6 for ALT treated groups.
