# Supplementary material for: Targeting the ICOS/ICOS‐L pathway in a mouse model of established allergic asthma disrupts T follicular helper cell responses and ameliorates disease
Source: Allergy. 2018 Nov 12;74(4):650–62. doi: 10.1111/all.13602 (PMC6492018; doi:10.1111/all.13602)
Supplement: Supplementary file 2 [file ALL-74-650-s002.docx]

**SUPPLEMENTARY METHODS**

***Tissue Processing:***

Lung cells were disaggregated by incubating chopped tissue at 37 ˚C for 45 minutes in complete media (RPMI with 10% fetal calf serum, 2 mM L-glutamine and 100U/ml Penicillin/Streptomycin) containing 0.15 mg/ml collagenase type D (Roche Diagnostics, Burgess Hill, UK) and 25 µg/ml DNase type 1 (Roche Diagnositics). Splenic, mediastinal lymph node (mLN) and lung cells were recovered by filtering through a 100 µM nylon sieve and washing in complete media. Lung and splenic cells were then treated with red blood cell lysis buffer (155 mM ammonium chloride, 10 mM potassium bicarbonate and 0.1 mM Disodium EDTA) for 5 mins, washed and resuspended in complete media. Bronchoalveolar lavage (BAL) was collected by washing the airways three times with 0.4ml PBS via a tracheal cannula. BAL cells were pelleted and resuspended in 0.5ml complete media. Viable cells were counted by haemocytometer using trypan blue exclusion. Serum was acquired by collecting blood from a peripheral artery using Na-EDTA coated capillaries and centrifuging at 12,000 g for 15 mins.

***MCPT1 measurement:***

MCPT1 was measured using a Ready Set Go Kits (eBioscience, CA, USA) according to manufacturer’ instructions.

***Histological assessment:***

Paraffin embedded formalin fixed lung lobes were sectioned and subjected to Periodic Acid-Schiff (PAS) and picro sirus red staining. Sections were scored and assessed as previously described^1^.

***Total Collagen:***

Total collagen was assessed in lung homogenate using the Sircol^TM^ Soluble Collagen assay (Biocolor, Carrickfergus, UK) according to manufacturer’s instructions.

***Airway smooth muscle assessment:***

Paraffin embedded formalin fixed lung lobes were sectioned and immunohistochemically stained with rabbit anti-mouse smooth muscle actin (Abcam, Cambridge, UK) as previously described^2^. Lung sections were imaged at x20 magnification and the size of the airway smooth muscle layer was measured. Briefly, the thickness of 10 airways were measured per mice and 10 measurements were taken per airway. The data is presented as an average. Data was blinded for analysis.

**SUPPLEMENTARY FIGURE LEGENDS**

**Table S1: Flow cytometry antibodies used in the study**

Lineage negative (Lin^-^) cells were defined as: CD11b^-^CD19^-^TCR-β^-^TER119^-^GR1^-^CD3^-^CD4^-^CD8^-^

**Figure S1: T follicular helper cell gating strategy**

T follicular helper cells (T_FH_) were identified by flow cytometry and defined as CXCR5^+^PD1^+^Foxp3^-^CD44^hi^D62L^-^CD4^+^CD8^-^CD3^+^ lymphocytes.

**Figure S2: T_FH_ are induced in the spleen but not in the circulation following allergen exposure.**

Adult female BALB/c mice were exposed to either 25 µg house dust mite (HDM), 10 µg *alternaria alternata (*ALT) or 25 µl phosphate buffered saline (PBS), 3 times a week for up to 5 weeks. Flow cytometry was used to determine the frequency of T_FH_ within cellular compartments following allergen exposure. T_FH_ were defined as CXCR5^+^PD1^+^Foxp3^-^CD4^+^. Representative flow plots of T_FH_ in PBS, ALT or HDM treated animals are displayed, pre-gated on CD4^+^CD3^+^Foxp3^-^CD44^hi^CD62L^-^ lymphocytes. Data is quantified. **A)** Spleen, **B)** Blood. Statistical significance was determined using a Mann Whitney U test. * *P<0.05*, ***p<0.01*, ****p<0.001*, n=5 per time-point. Representative data from 2 independent experiments.

**Figure S3: Germinal centre B cell gating strategy**

Germinal centre B cells were identified by flow cytometry and defined as CD19^+^B220^+^IgD^-^IgM^-^CD38^-^GL7^+^FAS^+^ lymphocytes.

**Figure S4: GC B cells accumulate over time following allergen exposure.**

Adult female BALB/c mice were exposed to either 25 µg house dust mite (HDM), 10 µg *alternaria alternata (*ALT) or 25 µl phosphate buffered saline (PBS), 3 times a week for up to 5 weeks. Flow cytometry was used to determine the frequency of germinal centre (GC) B cells within cellular compartments following allergen exposure. GC B cell were defined as CD38^-^GL7^+^FAS^+^CD19^+^B220^+^ cells Representative flow plots of GC B cells in PBS, ALT or HDM treated animals, pre-gated on CD19^+^B220^+^ lymphocytes. **A)** Mediastinal lymph node (mLN), **B)** Lung, **C)** Spleen. Representative data from 2 independent experiments.

**Figure S5: ICOS/ICOS-L interactions reduces serum MCPT1.**

Adult female BALB/c mice were exposed (i.n) to 25 µg house dust mite (HDM) or 25 µl phosphate buffered saline (PBS), 3 times a week for 5 weeks. From the start of week 4 mice were also administered 150 µg anti-ICOS-L (α-ICOS-L) or isotype control (IgG) antibody (i.p) 3 times a week. Mice were culled at the end of week 5. **A)** Serum MCPT1 was measured by ELISA. Statistical significance was determined using a Mann Whitney U test. * *P<0.05*, ***p<0.01*, ****p<0.001.* Data is from 1 study, n=4 for PBS treated groups, n=6 for HDM treated groups.

**Figure S6: ICOS signalling is required to sustain T_FH_ during ALT driven chronic AAD.**

Adult female BALB/c mice were exposed (i.n) to 10 µg alternaria alternata (ALT) or 25 µl phosphate buffered saline (PBS), 3 times a week for 5 weeks. From the start of week 4 mice were also administered 150 µg anti-ICOS-L (α-ICOS-L) or isotype control (IgG) antibody (i.p) 3 times a week. Mice were culled at the end of week 5. **A)** Schematic of experimental design, **B)** Representative flow plots of mLN and lung T_FH_ following ALT and IgG or α-ICOS-L treatment. The data is quantified for all groups. **C)** Representative flow plots of mLN and lung germinal centre (GC) B cells following ALT and IgG or α-ICOS-L treatment. Pre-gated on CD19^+^B220^+^ B cells. **D)** Representative flow plots of mLN and lung B220^-^CD138^+^ plasmablasts following ALT and IgG or α-ICOS-L treatment, pre-gated on lymphocytes. Plasmablast numbers are quantified for all groups. Statistical significance was determined using a Mann Whitney U test. * *P<0.05*, ***p<0.01*, ****p<0.001.* Experiment was performed once. n=4 for PBS treated groups, n=6 for ALT treated groups.

**Figure S7: ICOS-L blockade reduces allergen specific IgE.**

Adult female BALB/c mice were exposed (i.n) to 10 µg alternaria alternata (ALT) or 25 µl phosphate buffered saline (PBS), 3 times a week for 5 weeks. From the start of week 4 mice were also administered 150 µg anti-ICOS-L (α-ICOS-L) or isotype control (IgG) antibody (i.p) 3 times a week. Mice were culled at the end of week 5. A) Serum was titrated and allergen specific antibody was measured by ELISA. Endpoint titres are displayed for IgE and IgG1, B) Serum MCPT1 was determined by ELISA. Statistical significance was determined using a Mann Whitney U test. * *P<0.05*, ***p<0.01*, ****p<0.001.* Experiment was performed once. n=4 for PBS treated groups, n=6 for ALT treated groups.

**Figure S8: Therapeutic ICOS-L blockade improves chronic allergic airway disease.**

Adult female BALB/c mice were exposed (i.n) to 10 µg alternaria alternata (ALT) or 25 µl phosphate buffered saline (PBS), 3 times a week for 5 weeks. From the start of week 4 mice were also administered 150 µg anti-ICOS-L (α-ICOS-L) or isotype control (IgG) antibody (i.p) 3 times a week. Mice were culled at the end of week 5. A) Number of lung cells, B) Lung eosinophil numbers, C) Proportions of lung eosinophils, D) Airway hyperresponsiveness was measured by exposing mice to 0-100mg/ml methacholine (MCh) using the flexiVent system during ALT induced allergic airway disease. Airway resistance, elastance and compliance were measured. Curves display mean±SEM. Statistical significance between ALT+ IgG and ALT+α-ICOS-L groups was determined using a Mann Whitney U test. * *P<0.05*, ***p<0.01*, ****p<0.001. .* Experiment was performed once. n=4 for PBS treated groups, n=6 for ALT treated groups.

**Figure S9: Therapeutic ICOS-L blockade does not alter mucus hyper-secretion, collagen deposition or airway smooth muscle thickening.**

Adult female BALB/c mice were exposed (i.n) to 25 µg house dust mite (HDM), 10 µg *alternaria alternata (*ALT) or 25 µl phosphate buffered saline (PBS), 3 times a week for 5 weeks. From the start of week 4 mice were also administered 150 µg anti-ICOS-L (α-ICOS-L) or isotype control (IgG) antibody (i.p) 3 times a week. Mice were culled at the end of week 5. **A-B)** Representative images of Periodic Acid-Schiff (PAS) staining on parafilm embedded formalin fixed (PEFF) lung sections used to score mucus hyper-secretion. **A)** HDM+ IgG**, B)** HDM+α-ICOS-L**, C)** Quantification of PAS scoring. **D-E)** Representative staining of Pico-Sirus Red on PEFF lung used to determine collagen I and III deposition. **D)** HDM+ IgG**, E)** HDM+α-ICOS-L, **F)** Total collagen in the lungs determined by Sircol soluble collagen assay. **G-H)** Representative staining of airway smooth muscle cells and myofibroblasts identified by immunohistochemical staining of α-smooth muscle actin (α-SMA). **G)** HDM+ IgG**, H)** HDM+α-ICOS-L, **I)** Airway smooth muscle layer thickness was measured from α-SMA stained sections. Images were taken at x20 magnification. Statistical significance was determined using a Mann Whitney U test. Data is pooled from two independent experiments, n=8 for PBS treated groups, n=12

**Figure S10: IL-13^+^ CD4^+^ T cells are reduced by ICOS-L blockade in alternaria alternate driven allergic airway disease.**

Adult female BALB/c mice were exposed to either 25 µg House dust mite (HDM), 10 µg *alternaria alternata (*ALT) or 25 µl phosphate buffered saline (PBS), 3 times a week for up to 5 weeks. From the start of week 4 mice were also administered 150 µg anti-ICOS-L (α-ICOS-L) or isotype control (IgG) antibody (i.p) 3 times a week. Mice were culled at the end of week 5. Flow cytometry was used to determine the frequency of lung cellular populations. **A-B)** Lung ILCs were defined as Lin^-^Nkp46^-^CD45^+^CD90.2^+^ and the proportion and total number were calculated, **B)** HDM study, **C)** ALT study. **C)** Representative gating of IL-13^+^ CD4^+^ T cells and IL-13^+^ ILC2s following HDM and IgG or α-ICOS-L treatment. Data is quantified for all groups. **D)** Proportions of lung IL-13^+^ T cells and ILCs. **E)** Numbers of lung IL-13^+^ T cells and ILCs. Experiment was performed once. n=4 for PBS treated groups, n=6 for ALT treated groups.

**Figure S11: IL-17A^+^ CD4^+^ T cells are not directly targeted by ICOS-L blockade**

Adult female BALB/c mice were exposed to either 25 µg house dust mite (HDM), 10 µg *alternaria alternata (*ALT) or 25 µl phosphate buffered saline (PBS), 3 times a week for up to 5 weeks. Flow cytometry was used to determine the frequency of lung cellular populations. **A)** Representative gating of IL-17A^+^ CD4^+^ T cells and IL-17A^+^ ILCs following allergen and IgG or α-ICOS-L treatment. These populations were quantified **B)** Proportions of lung IL-17A^+^ CD4^+^ - HDM study, **C)** Number of lung IL-17A^+^ CD4^+^ - HDM study**, D)** Proportions of lung IL-17A^+^ CD4^+^ - ALT study, **E)** Number of lung IL-17A^+^ CD4^+^ - ALT study, **F)** Proportions of lung IL-17A^+^ ILCs - HDM study, **G)** Proportions of lung IL-17A^+^ ILCs - ALT study, **H)** Number of lung IL-17A^+^ ILCs - HDM study, **I)** Number of lung IL-17A^+^ ILCs - ALT study, Statistical significance was determined using a Mann Whitney U test. * *P<0.05*, ***p<0.01*, ****p<0.001.* HDM data is pooled from two independent experiments, n=8 for PBS treated groups, n=12 for HDM treated groups. ALT experiment was performed once, n=4 for PBS treated groups, n=6 for ALT treated groups.

**Figure S12: Therapeutic ICOS-L blockade reduces T regulatory cells during Alternaria alternate driven allergic disease**

Adult female BALB/c mice were exposed (i.n) to 10 µg Alternaria Alternata (ALT) or 25 µl phosphate buffered saline (PBS), 3 times a week for 5 weeks. From the start of week 4 mice were also administered 150 µg anti-ICOS-L (α-ICOS-L) or isotype control (IgG) antibody (i.p) 3 times a week. Mice were culled at the end of week 5. Flow cytometry was used to determine the number of Foxp3^+^CD4^+^ T cells within the mLN and lungs. **A)** Representative flow cytometry of Foxp3^+^ cells in allergen treated mice given IgG or α-ICOS-L. Pre-gated on CD4^+^CD3^+^ T cells. **B)** Numbers of mLN and lung Foxp3^+^CD4^+^ T cells were quantififed. Statistical significance was determined using a Mann Whitney U test. * *P<0.05*, ***p<0.01*, ****p<0.001.* ALT experiment was performed once, n=4 for PBS treated groups, n=6 for ALT treated groups.

**REFERENCES**

1. Saglani S, Mathie SA, Gregory LG, Bell MJ, Bush A, Lloyd CM. Pathophysiological Features of Asthma Develop in Parallel in House Dust Mite–Exposed Neonatal Mice. *American Journal of Respiratory Cell and Molecular Biology* 2009; **41**(3)**:** 281-289.

2. Gregory LG, Mathie SA, Walker SA, Pegorier S, Jones CP, Lloyd CM. Overexpression of Smad2 drives house dust mite–mediated airway remodeling and airway hyperresponsiveness via activin and IL-25. *American journal of respiratory and critical care medicine* 2010; **182**(2)**:** 143-154.
